# “Cocoa and Chocolate: Science and Gastronomy”—The Second Annual Workshop of the Research Institute on Nutrition and Food Security (INSA): 9 November 2016

**DOI:** 10.3390/nu9020156

**Published:** 2017-02-17

**Authors:** Malen Massot-Cladera, Francisco Pérez-Cano, Rafael Llorach, Mireia Urpi-Sarda

**Affiliations:** 1 Department of Biochemistry and Physiology, Faculty of Pharmacy and Food Sciences, University of Barcelona, 08028 Barcelona, Spain; malen.massot@ub.edu (M.M.-C.); franciscoperez@ub.edu (F.P.-C.); 2 Department of Nutrition, Food Science and Gastronomy, Faculty of Pharmacy and Food Sciences, Campus Torribera, University of Barcelona, 08921 Santa Coloma de Gramanet, Spain; rafallorach@ub.edu

## 1. Preface

The Research Institute on Nutrition and Food Security at the University of Barcelona (INSA-UB) was founded in 2005 by twenty-two research groups from the Faculties of Pharmacy and Food Science; Biology; Chemistry; and Geography and History, as well as other UB-affiliated centers and hospitals. Most of the groups at the Institute are, or at least are part of, the research groups established by the Government of Catalonia. INSA-UB was founded to meet the current societal need for research, training and service provision in the sectors related to the agro-alimentary industry. Researchers at the Institute are experts in different fields of nutrition; food analysis and control; food safety and the study of the social and economic impact of food.

The main objectives of the institute are to promote research in the fields in which it works; to encourage collaboration between researchers and the establishment of multidisciplinary teams; to promote participation in research programs and institutional administration, particularly in European research projects; to encourage the development of joint projects with companies in the sectors related to its scope; to make available all the social potential of the UB in this area, especially the training of technicians and specialists and provision of services; to promote the transfer of knowledge and the dissemination of research results between society and government; and to advise consumers, businesses and public authorities on nutrition, food safety and quality.

The Second Annual Workshop hosted by INSA-UB titled, “Cocoa and Chocolate: Science and Gastronomy” provided a forum where the latest findings around three aspects relating to cocoa (gastronomy, food technology and nutritional health) could be disseminated, and to understand its complexity.

Cocoa is a product that originates from the beans of *Theobroma cacao*. It contains macronutrients, such as proteins, lipids and carbohydrates, micronutrients, and bioactive compounds, such as flavanols, fiber, and theobromine [1]. However, in addition to its nutritional characteristics, its consumption is associated with pleasure or with different emotional changes. The gastronomical interest in cocoa is increasing due to its sensory properties and use in new cuisine, as well as new tendencies towards high-priced chocolates and delicacies containing high amounts of cocoa. The chocolate industry and its market are large and wide due to cocoa products having reached a critical mass. Finally, in terms of nutrition and health, its bioactives (epicatechin and procyanidins) infuse cocoa with healthy properties, which can provide beneficial protection towards cardiovascular risk factors, brain and immune functions, and cancer prevention, among others [2,3,4,5,6].

The “Cocoa and Chocolate: Science and Gastronomy” workshop took place at the headquarters of INSA-UB, located at the Food and Nutrition Torribera Campus, University of Barcelona (Santa Coloma de Gramanet, Barcelona, Spain). Although the main invited scientific presenters focused on different aspects of cocoa and chocolate, the selected oral and poster presentations reflected the research related to the Institute on nutrition and food safety, and provided an opportunity to share research and create new partnerships with members of the Institute and participants from other institutions and/or industries. More than 150 delegates from 16 different institutions attended to the II Annual Workshop on 9 November 2016.

The workshop opened with the results of the research grant awarded in 2014 by INSA-UB. The different sessions focused on cocoa and chocolate, divided thematically into: (1) history and gastronomy; (2) science and technology; and (3) nutrition and health. The session regarding history and gastronomy as they related to cocoa and chocolate discussed some of the issues surrounding the worldwide importance of cocoa products in the market; the outlook of different cultures around chocolate; and how industry uses these concepts to exploit the sector and sell products. This session also discussed the use of chocolate throughout different historical periods and cuisines around the world. This last presentation provided a technological point of view and looked at the manner in which industry had used history to evolve from using a metate, the first tool used to produce chocolate, towards the development of new manufacturing processes to arrive at the current movement of “Bean to Bar”. Other technological approaches addressing the structures and textures of chocolate though sharp melting and the melting behavior of crystals to obtain new textures of chocolate were also discussed. The workshop also offered three different practical sessions focused on historical and new concepts of cocoa and chocolate. The first practical session used tempering chocolate to view the effects of crystallization in cocoa on the final quality of the chocolate coating. The second practical focused on the application of chocolate in the mythical creations of modern cuisine, where participants practiced formulating different textures of chocolate, including spherifications and foams. The third practical allowed participants to taste cocoa and cocoa products by its passage through history and different types of cultivation. The last part of the workshop highlighted the wide range of health effects attributed to cocoa consumption, including its positive effects on chronic diseases, cognitive function and cardiovascular disease, and emphasized the role of the main bioactive cocoa compounds responsible for these effects.

This Conference Report contains the summary of lectures (Section 2) and selected abstracts (Section 3) detailing in each subsection the title of the presentation followed by the author’s names. All the author’s affiliations are detailed in the appendix section at the final of the manuscript (Appendix A).

## 2. Summary of Lectures

### 2.1. The History and Consumption of Chocolate in Catalonia, Spain

GilF.

Did you know that the average person in Catalonia consumes 3.2 kg of chocolate per year, which is slightly above the Spanish average of between three and 3.1 kg, per person per year? In contrast, the consumption of chocolate in countries, such as Germany, Switzerland, Belgium, the USA and Japan is between eight and 13 kg, per person per year. What are the causes of this imbalance between some countries and others? Furthermore, did you know that 90% of the population worldwide likes chocolate?

Chocolate, therefore, has massive product acceptance, or as marketing experts’ say, “the product has a critical mass”. Only 10% of those asked stated that they did not like chocolate. Few products are as widely popular with consumers, a fact that should be exploited by the chocolate and cocoa product sector. However, despite being such a popular and universal product, there are many unknown elements for the immense majority of people.

Another important aspect to consider is the industrial point of view. In Spain, the entire chocolate and cocoa products industry has to appeal to the public to buy chocolate as part of their “gift” expenditure; while in France, the focus is based on one’s own consumption, gastronomic pleasure or the hedonistic factor (“I indulge myself”). This can be seen by the much higher levels of chocolate consumption in France than in Spain. As chocolate bars can be elements of socialization and thus become the undisputed leaders in the market for middle and upper-middle class people, it is the area a company wants to be managed effectively.

For instance, it is interesting to analyze the strategy used by one of the most important chocolate industries in Spain. Valor, a family business located in Villajoyosa with over 130 years of history, focuses on “WHO”, being understood as a variable response: “who may my consumers be” rather than on “WHAT”, being understood as the product on offer. This is called market-driven management or company management with market orientation.

### 2.2. Cooking with Chocolate: Catalan Cuisine

TresserrasJ.

Chocolate was introduced to European gastronomy from the colony of New Spain (specifically, from the territory that currently corresponds to Mexico and Central America). It expanded out of Spain thanks to monastic orders, and the fame that it acquired in the Spanish Court and at the tables of the nobility and the commercial bourgeoisie. Its basic consumption was as a beverage (taken in water and later in milk) that was sweetened (with honey or sugar), flavored (with vanilla, cinnamon, or other additives) and with different degrees of viscosity (watery or thick). Special tableware associated with its preparation and consumption was even designed. This consisted of the chocolate grinder; the jicaras in which the hot chocolate was served; and a tray called the mancerina (in honor of Pedro Álvarez de Toledo y Leiva, VI Marqués de Mancera and Viceroy of Peru who made it fashionable) where it was accompanied with puff pastry or pancakes. Its consumption became popular with the appearance of the first chocolatiers.

One of the characteristics of Baroque cuisine was the use of “table chocolate”, obtained by grinding of cocoa or chocolate with nuts and spices, in making sauces. Undoubtedly, the Mexican moles, especially the “poblano mole”, are the best-known chocolate-based sauces. In Europe, it was used in the preparation of chocolate sauces that basically served to accompany bush meat dishes. Catalan cuisine is one of a few European cuisines that incorporate chocolate in their sauces, not only for meat but also fish and seafood, as well as mixed “turf and surf” dishes.

In the nineteenth century, the solidification of chocolate led to the development of new processes that produced chocolate bars and chocolate sweets, and thus expanded its use in confectionery.

Today, a study on the use of chocolate in the traditional cuisine of the Catalan coast is being coordinated by the Catalan Coast Cuisine Museum, located in Can Ganga in Tossa de Mar (Costa Brava, Spain) and is managed by the town hall and the University of Barcelona.

### 2.3. Technology of Cocoa and Chocolate: From Cocoa to Chocolate

CollX.

This talk was given from the personal point of view of the managing director of a chocolate production facility. This lecture explained the process of manufacturing chocolate and its evolution from the “metate”, the first utensil used for its manufacture, to the current tendency: “Bean to bar”.

Major milestones in the evolution of the manufacturing process were introduced: from the great discoveries of the nineteenth century that allowed the popularization of the product; to its mass production in the second half of the twentieth century due to the development of mechanical technology and product sophistication; and the introduction of robotics at the end of the twentieth century. Finally, the incipient “return to origins” model was explained, which is driven in part by the new consumer values of the twenty-first century and the rapid development promoted by social networks.

### 2.4. Structures and Textures of Chocolate

Bayés-GarcíaL.Cuevas-DiarteM.A.CalvetT.

Chocolate is made up of cocoa butter crystals formed as a continuous body in which tiny particles of sugar, cacao mass, and other ingredients are dispersed. Sharp melting and the quick release of flavor and sweetness/bitterness are determined by the melting behavior of cocoa butter crystals. Cocoa butter exhibits six different polymorphic forms, referred to as I–VI. Among them, form V is industrially promoted through specific dynamic thermal treatments (tempering), as this polymorph provides the desired melting, textural, and mouth-feel characteristics of chocolate, so that this form may also be maintained over time. Nevertheless, some undesired external factors, such as storage at high temperatures, temperature fluctuation or oil migration processes may cause the blooming phenomenon, which is caused by the formation of form VI needle-shaped cocoa butter crystals and is of significant concern for the chocolate and confectionery industries. Dynamic thermal treatments may be also applied for the development of new chocolate textures, such as the velvet effect. Thermal treatments enable the formation of thin layers of cocoa butter crystals with much smaller particle sizes and a lower melting point compared to normally-tempered chocolate, which creates the soft-mouth feeling.

### 2.5. Health Implications of Regular Cacao Consumption

Bravo ClementeL.

Until recently, chocolate and cocoa were a most restrained pleasure. Their consumption was often linked to negative effects like increased blood lipids, weight gain, or skin problems. Scientific research over the last two decades; however, has depicted a very different scenario. Indeed, consuming cocoa products has shown positive effects on blood pressure; endothelial function; antioxidant status; platelet activity or insulin resistance; in addition to improvements in mood and cognitive function. These beneficial health effects have been attributed to the polyphenolic fraction of cocoa, which is rich in flavanols and procyanidins, since it is commonly accepted that a diet rich in flavonoids promotes health and delays the onset of different non-transmittable pathologies, such as cardiovascular disease, Type 2 diabetes mellitus, obesity, etc. However, other bioactive compounds in cocoa products such as methylxanthines and dietary fiber should not be overlooked. The author reviewed the scientific evidence for the beneficial effects of cocoa and chocolate consumption on some of the most common chronic diseases, looked at the mechanisms of action involved and considered the contribution of non-phenolic bioactive compounds.

### 2.6. Effects of Cocoa on Cardiovascular Health

EstruchR.

Evidence based on epidemiological studies suggests that dietary flavonoids may play a critical role in the prevention of coronary heart disease (CHD). Cocoa (*Theobroma cocoa*) and its derived products, such as cocoa powder, represent a very rich source of dietary flavonoids, containing a higher content per serving than tea, red wine, legumes or fruit. The health benefits associated with cocoa consumption have been related to their protective effect mainly on cardiovascular disease, but also in other related diseases such as diabetes and age-related cognitive decline. Observational studies have shown that the Kuna India population from the San Blas Islands of Panama has very low rates of hypertension and cardiovascular disease, effects that have been related to their high cocoa intake. Epidemiological and clinical trials have also evaluated the effects of cocoa intake on different cardiovascular risk factors. Several studies have confirmed that cocoa intake reduces blood pressure in normotensive and hypertensive subjects. In fact, dark chocolate intake increases nitric oxide (NO) generation that leads to vasodilatation and reduces systolic and diastolic blood pressure by 2.77 mmHg and 2.20 mmHg, respectively, whereas white chocolate does not reduce blood pressure. Another mechanism by which flavanols may lower blood pressure is through the inhibition of the angiotensin-converting enzyme (ACE). In addition, cocoa intake improves lipid profile and insulin sensibility; reduces platelet activity and function; and ameliorates endothelial dysfunction and arterial stiffness. At least part of these beneficial effects have been attributed the anti-inflammatory and anti-oxidant properties of the polyphenols contained in cocoa. Other beneficial effects may also be due to changes in gut microflora. Recent population-based studies have observed an inverse relationship between all-cause mortality, and cardiovascular morbidity and mortality. In conclusion, cocoa consumption ameliorates cardiovascular risk factors, including diabetes, and reduces cardiovascular morbidity and mortality. However, the beneficial effects may be even higher if cocoa consumption is included into a well-balanced healthy diet, such as the Mediterranean diet.

### 2.7. The Food Metabolome Biomarker: A New Strategy for Evaluating the Intake of Dietary Bioactive Compounds, Foods and Dietary Patterns. Summary of the Project FRI-2013

Urpi-SardaM.Farran-CodinaA.Andres-LacuevaC.LlobetJ.M.LlorachR.

The study of the relationship between dietary bioactive components (i.e., phytochemical) intake and health status requires accurate measurements of dietary exposure. Thus, although biomarkers cannot completely replace traditional methods of dietary assessment, there is broad consensus that the application of Metabolomics in the detection and identification of novel and robust biomarkers of exposure to diet and/or consumption of healthy foods can improve and validate these methods.

The main aim of this project was to define the technical and scientific aspects of “Food Metabolome” as a new strategy for identifying biomarkers that allow the evaluation of exposure to healthy diets.

A pilot study was carried out with 45 students who twice collected 24 h urine samples. In addition, a validated food frequency questionnaire and three-day food recall was obtained from each participant. Differences within the information provided by the traditional methods of collecting dietary information were analyzed with respect to the food metabolome. The composition of the food metabolome was compared according to the stratification of the population based on the consumption of foods or dietary patterns. The relevance and originality of this project lies in merging the new powerful nutrimetabolomic fingerprint approach with well-known dietary epidemiology techniques to produce new insights into the biomarkers of healthy dietary intake patterns in a student population under free-living conditions.

## 3. Selected Abstracts

In this section, the abstracts were presented orally or as a conference poster. A total of five oral presentations and 26 conference posters were selected for inclusion.

### 3.1. Oral Presentations

#### 3.1.1. Histamine Intolerance Management in Clinical Practice: Do Putrescine Contents Justify the Exclusion of Certain Foods?

Comas-BastéO.Latorre-MoratallaM.L.Veciana-NoguésM.T.Vidal-CarouM.C.

***Background and objectives*:** Histamine intolerance is a disorder in the homeostasis of histamine due to reduced enzymatic intestinal activity, which causes an accumulation of this amine in plasma and the appearance of multi-faced allergy-like clinical symptoms. Current clinical strategy treatments are based on the limitation of histamine containing food and/or the supplementation with exogenous diamine oxidase enzyme (main enzyme of histamine intestinal metabolism). Histamine distribution in food is wide and variable, even among different batches of the same product. However, some foods (presumably without or with small amounts of this amine) are usually excluded from these restrictive diets for being related to the onset of symptoms. The presence of other bioactive amines such as putrescine, could be co-responsible for triggering adverse effects by competing for the same metabolic pathway. The aim of this work was to study the occurrence of putrescine in some foods of plant origin where their exclusion was not justified by their histamine content. Batch origin, storage conditions (one week at four degrees Celsius) and peel removal were studied in order to assess their contribution to putrescine content.

***Methodology*:** Three vegetables, courgette (*n* = 24), paprika (*n* = 10) and cucumber (*n* = 9), were selected out of the group of totally excluded vegetal products recommended by the clinical management of histamine intolerance, despite the presumable absence of histamine. The content of histamine and putrescine in these items were determined in triplicate by ultra-high-pressure liquid chromatography with fluorescence detection (UHPLC-FL).

***Results and conclusions*:** Histamine was not found in any of the analyzed products. Putrescine was present in all samples of courgette (ranging from three to 13 mg/kg); paprika (one to 145 mg/kg); and cucumber (one to seven mg/kg). In general, there were no statistically significant differences in putrescine content among products in the same batch; however, those differences were significant when different origins were considered. The elimination of the courgette and cucumber peels did not influence putrescine content. A significant increase in putrescine levels was observed in practically all samples subjected to one-week refrigerated storage, with an average increase up to 53% in the case of courgette. Although the absence of histamine in courgette, paprika and cucumber has been confirmed, putrescine found in these products could potentially explain their association with the symptomatology of histamine intolerance as it competes with histamine detoxifying enzymes. The increase of putrescine during domestic storage also needs to be taken into account, and product freshness is a key parameter to be considered in minimizing putrescine intake in the dietary framework of histamine intolerant patients.

#### 3.1.2. Home Cooking and Ingredients Synergism Improve Lycopene Isomers in *Sofrito*

AlvarengaJ.F.R.TranC.Hurtado-BarrosoS.Martínez-HuélamoM.IllanM.Lamuela-RaventósR.M.

***Background and objectives*:** Tomato products rich in lycopene *Z*-isomers are of interest since these carotenoids present more bioavailability and antioxidant capacity than the all-E lycopene forms. Intrinsic food properties, processing, and the interaction between dietary components are all factors that can influence the content, type and bioavailability of carotenoids. The aim of this study was to evaluate how the content of carotenoids and their isomerization in tomato-based Mediterranean *sofrito*, might be affected by the process of home cooking, as well as by the presence of other ingredients.

***Methodology*:** A full factorial design 24 was applied to clarify the contribution of extra virgin olive oil (5%–10%), onion (20%–40%), and garlic (2%–4%); and cooking duration (30–60 min) on the carotenoid composition of *sofrito*. The identification of the carotenoids was based on retention time; chromatography with standards; UV/VIS absorption spectrum: λmax, %III/II and %Ab/II; and mass spectrum. High pressure liquid chromatography with diode array detection (HPLC-DAD) was used to provide quantitative results, in conjunction with external calibration curves with standards.

***Results and conclusions*:** The main factors associated with a higher production of 5-*Z*-lycopene, 9-*Z*-lycopene and 13-*Z*-lycopene in *sofrito* were the cooking duration and onion content. Onion proved to be the most interesting ingredient in the *sofrito* formulation due to its effect on enhancing lycopene isomerization. This vegetable could be explored as an ingredient to improve the bioavailability of lycopene in tomato products.

**Acknowledgments:** National Council for Scientific and Technological Development (CNPq, Brazil); The Ministry of Education, Culture and Sport (MECD), Spanish Ministry of Economy and Competitiveness (MEC), CIBER Physiopathology of Obesity and Nutrition (CIBEROBN) and Generalitat de Catalunya (Spain).

#### 3.1.3. Biomarkers of Cocoa Intake: Multimetabolite Biomarker Models as a Novel Strategy to Improve Dietary Assessment

Garcia-AloyM.LlorachR.Urpi-SardaM.Vázquez-FresnoR.JaureguiO.Andres-LacuevaC.

***Background and objectives*:** There is a growing body of evidence on the beneficial effects of cocoa consumption on the cardiovascular system. Untargeted metabolomics was used as a hypothesis-generating tool.

The aim of this work was to contribute to the identification of biomarkers related to cocoa ingestion (biomarkers of intake).

***Methodology*:** An untargeted high-pressure liquid chromatography-time of flight mass spectrometry (HPLC-Q-ToF-MS) metabolomics strategy was applied in urine samples collected in acute and short-term clinical trials, as well as in observational studies. To improve the prediction of cocoa consumption, a combined urinary metabolite model was constructed using stepwise logistic regression analysis. Receiver operating characteristic (ROC) curves were performed to evaluate the predictive capacity of candidate biomarkers.

***Results and conclusions*:** Dietary cocoa fingerprinting was characterized by using a complex metabolic pattern linked to cocoa phytochemicals (alkaloids and polyphenols) and processing-derived compounds. A large proportion of metabolites were characteristic of cocoa exposure independently of the study design. The area under the curve (AUC) values (95% confidence interval (CI)) for the model were 95.7% (89.8%–100%) and 92.6% (81.9%–100%) in the training and validation sets, respectively, whereas the AUC for individual metabolites were <90%.

Discriminating metabolites of cocoa exposure were replicated among three studies with different design, increasing the level of evidence from observed associations. The predictive capacity of dietary exposition was improved using multimetabolite combined models compared individually with the same compounds.

**Acknowledgments:** This work has been supported by Spanish Ministry of Economy and Competitiveness (MINECO) and co-founded by the Spanish Federation of Rare Disease FEDER: AGL2009-13906-C02-01; the CONSOLIDER-INGENIO 2010 Program, FUN-C-FOOD (CSD2007-063); PCIN-2014-133; and the PI13/01172 Project (Plan Nacional de I+D+i 2013–2016) by the “ISCIII-Subdirección General de Evaluación y Fomento de la Investigación”. We also thank the award of 2014SGR1566 from the Agency for Management of University and Research Grants (AGAUR). Mar Garcia-Aloy thanks the AGAUR for the predoctoral FI-DGR 2011 fellowship. Rosa Vazquez-Fresno thanks the Training of Research Staff FPI fellowship; Rafael Llorach and Mireia Urpi-Sarda thank the “Ramón y Cajal” program (RYC-201007334 and RYC-2011-09677, respectively), all from the MINECO and Fondo Social Europeo.

#### 3.1.4. Cocoa Consumption and Its Health Impact in University Students

VicenteF.Saldaña-RuízS.RabanalM.Rodríguez-LagunasM.J.PereiraP.Pérez-CanoF.J.CastellM.

***Background and objectives*:** Although cocoa is well recognized as an excellent source of polyphenols with multiple benefits in human health; and has shown immunomodulatory actions in rats, there are no observational data relating to cocoa consumption and immune diseases.

The aim of the present study was to associate cocoa consumption, obtained through a validated food frequency questionnaire (FFQ), with several health indicators in university students.

***Methodology*:** A sample of 270 university students from the University of Barcelona and Egas Moniz Cooperativa de Ensino Superior completed a validated FFQ for cocoa consumption [7] and a survey about their health status. The values of these health variables were then compared against students grouped according to their low (L), moderate (M) and high (H) cocoa consumption.

***Results and conclusions*:** University students had an average consumption of cocoa of about 13.2 g/day, mostly derived from chocolate bars, but also from dairy products. The proportion of people suffering chronic diseases was lower in the M group than in the L group. Likewise, the proportion of students with flu was lower in group H than those in the L and M groups. More importantly, the proportion of students with allergies was much lower in the M and H groups than those in the L group, with moderate cocoa intake associated with a lower presence of allergic symptoms.

**Acknowledgments:** The authors would like to thank Joan Vila from the Hospital del Mar Medical Research Institute (IMIM) for the technical and scientific support in data processing.

#### 3.1.5. The Effects of *Sofrito* on FGF21 (Fibroblast Growth Factor 21) Expression and Signaling

SandovalV.García-GuaschM.T.RodríguezR.RosellC.Lamuela-RaventósR.M.MarreroP.F.HaroD.RelatJ.

***Background and objectives*:** Obesity is a worldwide health problem due to its associated comorbidities. Fibroblast growth factor 21 (FGF21) is a hormone considered to be a promising therapeutic candidate for the treatment of obesity, and in the maintenance of metabolic homeostasis in healthy people. Previously published data suggests the exertion of FGF21 pleiotropic function on glucose/lipid metabolism.

Some polyphenols or polyphenol-rich foods modulate FGF21 expression. Our hypothesis was that FGF21 could link dietary polyphenols with their healthy metabolic effects. Accordingly, our objective was to study the effects of *sofrito* on FGF21 function in obese rats.

***Methodology*:** Lean and obese Zucker rats were fed for eight weeks with a *sofrito*-supplemented diet. Serum and different tissues were collected after sacrifice, and FGF21 expression and signaling were analyzed. The expression of FGF2; the FGF21 receptors (FGFR1 and FGFR4); the co-receptor KLB; and the mRNA levels of the FGF21 target genes c-fos and Egr1, were analyzed in liver and white adipose tissue (WAT).

***Results and conclusions*:** Obese individuals demonstrate FGF21 resistance as they have increased levels of FGF21 due to its overexpression in the liver in response to a down regulation of receptor expression in target organs. Our results reproduced this FGF21 resistance in obese rats; this phenotype was partially reverted in obese rats fed with a *sofrito*-supplemented diet shown by an induction of FGFR1 and KLB mRNA levels in visceral WAT. A down regulation of FGF21 expression in liver that correlated with increased mRNA levels of c-fos and Egr1 was also noted. Thus, the results suggested that a *sofrito*-supplemented diet improved FGF21 signaling in obese Zucker rats.

**Acknowledgments:** Ministerio de Educación, Becas The National Commissions for Scientific and Technological Research (CONICYT-Chile); SAF2013-41093, AGL2010-22319-C03; AGL2013-49083-C3-1-R from Spanish Ministry of Economy and Competitiveness; Instituto de Salud Carlos III (ISCIII), CIBER Physiopathology of Obesity and Nutrition (CIBEROBN).

### 3.2. Poster Sessions

#### 3.2.1. The Evaluation of Anti-Inflammatory Activity Using Zebrafish Larvae and Its Application to Compounds in Food

ArteagaC.BoixN.LlobetJ.M.Gómez-CatalánJ.

***Background and objectives*:** Inflammation is a normal protective response of the innate immune system to tissue injury. Based on the principle of generating a mechanical injury to attract leukocytes to damaged zones, zebrafish embryos and larvae have proven to be suitable for the investigation of the kinetics of inflammation in vivo. The principal aim of this work was to develop an experimental zebrafish larvae model to evaluate inflammatory activity quickly and efficiently, as well as the application of this model in order to study the anti-inflammatory activity of compounds present in foods.

***Methodology*:** Inflammation was induced after four days post-fertilization in zebrafish larvae by tail transection and incubation with bacterial lipopolysaccharides in order to obtain leukocyte migration to the zone of injury. Migrating zebrafish leukocytes were detected in situ by myeloperoxidase staining and image analysis.

***Results and conclusions*:** Optimal parameters (exposure time; temperature; image analysis; and quantification of migration) were established to evaluate anti-inflammatory activity. Using our optimized zebrafish larvae assay, we found that compounds with a well-known anti-inflammatory activity (indomethacin, doxepin, piroxicam, dibenzoidolium DPI) significantly inhibited migration. This effect was also observed for some natural substances such as naringenin, beta-carotene, oleuropein, quercetin-3-beta-glucoside, malvidin-beta-glucoside and kaempferol-3-beta-glucoside.

Finally, our data demonstrated that zebrafish larvae provide a rapid and reliable model in which to quantify neutrophil migration in vivo. We further proposed that this model might prove useful for screening the anti-inflammatory activity of natural substances.

**Acknowledgments:** This research was funded by Spanish Ministry of Economy and Competitiveness AGL2013-49083-C3-1-R. Scholarship granted by the Ecuadorian government through the National Secretariat for Higher Education, Science, Technology and Innovation (SENESCYT) program.

#### 3.2.2. In Vivo Screening of Probiotics and Prebiotics Potential on a Model of Acute Gastroenteritis by Rotavirus

Azagra-BoronatI.Rigo-AdroverM.M.Massot-CladeraM.Saldaña-RuizS.Rodríguez-LagunasM.J.CastellM.FranchÀ.Pérez-CanoF.J.

***Background and objectives*:** Group A rotaviruses are the most common causative agents of acute gastroenteritis in children under two years old. Previous reports have suggested breastfeeding and the use of probiotics and prebiotics as protective agents for ameliorating the clinical course of rotavirus infection. This study was designed to establish the utility of the suckling rat rotavirus infection model to evaluate the protective role of probiotics and prebiotics.

***Methodology*:** From the first day of life, Lewis neonatal suckling rats received daily particular strains of probiotics and prebiotics. At the end of the first week of life, the heterologous simian rotavirus SA11 was inoculated orally in the supplemented groups and in the non-supplemented group. Rotavirus infection was evaluated daily by clinical indexes based on color, texture and the amount of feces obtained. Fecal samples were used to quantify viral shedding and specific immunity by means of antibody production. Other variables, such as intestinal architecture impairment, changes in gut permeability, and gene expression of particular molecules in the intestine, were also evaluated.

***Results and conclusions*:** Two different probiotics and prebiotics were tested to study their differential impact. The behaviors of all strains and prebiotic molecules assessed differed substantially, from the protection of diarrhea to scarce effects. Furthermore, the mechanisms involved in such effects were also shown to be product-specific. In conclusion, these results demonstrated the suitability of this model for the screening of supplements like probiotics and prebiotics as anti-infective agents in the case of rotavirus acute gastroenteritis and for the investigation of their mechanisms of action.

#### 3.2.3. Impact of Cocoa Diet on the Gut Microbiota in a Rat Oral Sensitization Model

Camps-BossacomaM.Pérez-CanoF.J.FranchÀ.CastellM.

***Background and objectives*:** It is well known that there exists an interrelation among dietary compounds, microbiota and food allergies. Previous studies have reported that cocoa intake produces changes on intestinal microbiota and induces immune tolerance in a rat oral sensitization model. Taking these facts into consideration, our aim was to investigate the modifications on gut microbiota from a cocoa-enriched diet in an oral sensitization model.

***Methodology*:** Lewis rats were orally sensitized with ovalbumin plus cholera toxin and were fed either a 10% cocoa diet or a standard diet. Fecal microbiota was analyzed through a metagenomic study after four weeks of the dietary intervention.

***Results and conclusions*:** Gut microbiota did not undergo many changes due to the oral sensitization but showed differences in a cocoa-enriched diet. In this sense, the cocoa diet reduced the absolute abundance of bacteria from Firmicutes and Proteobacteria phyla, and increased that of Tenericutes and Cyanobacteria phyla. In particular, there was an increase in bacteria belonging to the RF39 order (Mollicutes class, Tenericutes phylum) and those belonging to the Streptophyta order (Chloroplast class, Cyanobacteria phylum). Even though no changes were observed in the absolute abundance of the Bacteroidetes phylum, cocoa intake increased the relative abundance of the *Prevotella* genus and *Bacteroides uniformis*.

In conclusion, a cocoa diet inhibited oral sensitization and modified the gut microbiota in a rat oral sensitization model, suggesting that these changes in microbiota composition might be partially responsible for the tolerogenic effect of cocoa.

**Acknowledgments:** This study was financially supported with funding from the Spanish Ministry of Economy and Competitiveness (AGL2011-24279).

#### 3.2.4. Diet and Sleep in Spanish Children and Adolescents with Attention Deficit Disorder

Carpio-AriasT.Ríos-HernándezA.AldaJ.A.Farran-CodinaA.Izquierdo-PulidoM.

***Background and objectives*:** Nutrient deficiencies and unhealthy diets have been related to attention-deficit/hyperactivity disorder (ADHD). Sleep disorders also influence ADHD symptoms. To date, little is known about the impacts of diet and sleep on ADHD symptoms. The aim of this study was to assess the relationship between diet and sleep disturbances in a sample of children and adolescents with ADHD.

***Methodology*:** A total of 120 children and adolescents (60 newly diagnosed with ADHD, and 60 controls) were studied in a sex- and age-matched case-control study. ADHD diagnosis was conducted by trained psychiatrists in accordance with the Diagnostic and Statistical Manual of Metal Disorders (DSM-IV-TR). Food consumption and nutrient intake (measured by a food frequency questionnaire) and sleep (measured with the sleep disturbance scale for children, a sleep diary and actigraphy) were objectively measured. Lineal regression was used to determinate associations between diet and sleep.

***Results and conclusions*:** Sleep disorders were more prevalent in ADHD children compared to the control group. It was found that a lower consumption of fiber, vegetable protein, thiamin, vitamin D, iron, folate, potassium, and magnesium was associated with a higher prevalence of total sleep problems (*p* < 0.05). In the same way, a lower consumption of fiber, vegetable protein, thiamin, vitamin B6, vitamin E, iron, folate, magnesium and zinc intake was associated with a higher prevalence of “Excessive Daytime Sleepiness” (*p* < 0.05). Our results are in accordance with other authors who concluded that nutritional deficiencies such as that of B vitamins, magnesium and zinc may impair sleep by altering neural responses via circulating intestinal hormones (e.g., insulin, CCK ghrelin) or by affecting the synthesis of serotonin and melatonin.

**Acknowledgments:** All phases of this study were supported by grant PI11/2009 from the Instituto de Salud Carlos III, Ministerio de Ciencia e Innovación, Spain; the Consejo Nacional de Ciencia y Tecnología (CONACYT-México); and the Consejo Nacional de Ciencia Tecnología e Innovación (SENESCYT-Ecuador).

#### 3.2.5. Trans-Lycopene from Tomato Juice Attenuates Immune-Inflammatory Biomarkers: A Dose-Response Intervention Trial

Colmán-MartínezM.Martínez-HuélamoM.Valderas-MartínezP.Arranz-MartínezS.Almanza-AguileraE.CorellaD.EstruchR.Lamuela-RaventósR.M.

***Background and objectives*:** The aim was to evaluate the effects of carotenoids from tomato juice (TJ) on the expression of inflammatory biomarkers, by performing a four-week dose-response nutritional trial in a population at high cardiovascular risk.

***Methodology*:** The study was an open, prospective, randomized, cross-over, and controlled clinical trial. Twenty-eight volunteers (mean age 69.7 ± 3.1 years; mean BMI 31.5 ± 3.6 kg/m^2^) at high cardiovascular risk were assigned to consume daily for four weeks in random order: 200 mL (low-dose, LD) or 400 mL (high-dose, HD) of TJ made with 5% common olive oil, or water as a control (C). Blood samples were collected at baseline (B) and after each intervention. Endpoints included changes in plasmatic carotenoids; pro-inflammatory cytokines C-reactive protein (CRP) and interferon-γ (IFN-γ); chemokines interleukin-8 (IL-8), eotaxin, and CXCL motif chemokine 10 (CXCL10); inter-cellular adhesion molecule 1 (ICAM-1); and vascular-cell adhesion molecule 1 (VCAM-1).

***Results and conclusions*:** Compared with the control group, both TJ interventions induced significant decreases in ICAM-1, VCAM-1, CRP, and IL-8 (*p* ˂ 0.05), and also showed a trend to reduce eotaxin, IFN-γand CXCL10, in a dose dependent manner. These decreases were significantly correlated, mainly with the *trans* isomeric form of lycopene, while the other carotenoids present in TJ were not associated with any significant changes in these molecules.

**Acknowledgments:** This work was supported by the Interagency Council on Science and Technology (CICYT) (AGL2010-22319-C03; AGL2010-22319-C02; AGL2010-22319-C01; AGL2013-49083-C3-1-R) and the Instituto de Salud Carlos III (ISCIII), CIBER Physiopathology of Obesity and Nutrition (CIBEROBN) from the Spanish Ministry of Economy and Competitiveness (MEC) and Generalitat de Catalunya (GC) 2014 SGR 773. Mariel Colmán Martínez thanks the University, Research and Information Society Department of the Generalitat de Catalunya (FI-DGR 2013); Miriam Martínez Huélamo would like to thank the Spanish Ministry of Science and Innovation (MICINN) predoctoral program; Palmira Valderas-Martínez thanks the trainee research staff grant (APIF) predoctoral fellowship from the University of Barcelona, and Sara Arranz thanks the “Sara Borrell” postdoctoral program (CD10/00151).

#### 3.2.6. Validation of a UHPLC-FL Method for the Determination of Histamine and Methylhistamine in Urine: A New Approach for the Diagnosis of Histamine Intolerance

Comas-BastéO.Latorre-MoratallaM.L.Veciana-NoguésM.T.Vidal-CarouM.C.

***Background and objectives*:** Histamine intolerance is a disorder in the homeostasis of histamine due to the reduced intestinal activity of the diamine oxidase (DAO) enzyme that causes an accumulation of this amine in plasma and the appearance of adverse effects. A new approach for the diagnosis of this intolerance could be through the determination of histamine and its metabolites in urine. The aim of this work was to develop and validate a rapid method in which to unequivocally determine histamine and methylhistamine in human urine by Ultra High Performance Liquid Chromatography and Fluorimetric detection (UHPLC-FL).

***Methodology*:** Purification and concentration of 24 h urine samples were achieved by solid-phase extraction using a mixed cation exchange cartridge. Chromatographic separation was performed using a Waters Acquity™ UHPLC equipment with an Acquity UHPLC™ BEH C18 column coupled to a fluorimetric detector.

***Results and conclusions*:** The method provided a satisfactory linearity and chromatographic sensitivity with a detection limit lower than 0.035 mg/L and a quantification limit falling below 0.045 mg/L for both analyses. The precision, in terms of relative standard deviation, was lower than 5.5% and the accuracy, as mean recovery, was higher than 99% for both analyses. The UHPLC-FL method described has been demonstrated as a reliable procedure for determining histamine and methylhistamine in less than 11 min of chromatographic elution. The applicability of the method was studied in urine samples from volunteers, resulting in a reliable tool for the determination of both compounds and, is therefore a potential new approach for the routine diagnosis of histamine intolerance.

#### 3.2.7. Dietary Management of Histamine Intolerance: Influence of Putrescine in the Metabolism of Histamine by Diamine Oxidase Enzyme

Comas-BastéO.Latorre-MoratallaM.L.Veciana-NoguésM.T.Vidal-CarouM.C.

***Background and objectives*:** Histamine intolerance is a disorder in the homeostasis of histamine due to a reduced intestinal activity of diamine oxidase enzyme (DAO), which causes an accumulation of this amine in plasma. Current clinical strategies used for the treatment of this disorder are based on the exclusion of histamine containing foods and/or exogenous DAO enzyme supplementation. However, some foods without histamine or with low levels of this amine are excluded from these diets because patients relate them to the development of symptoms. Two hypotheses may be considered in order to explain this correlation: (a) certain foods can provoke the release of endogenous histamine; and (b) the presence of other amines, such as putrescine, in foods can trigger symptoms. In order to study this second hypothesis, the aim of this work was to evaluate the ability of putrescine to compete with histamine for the DAO enzyme. If putrescine was also metabolized by DAO, it could potentially increase the intestinal absorption of histamine and facilitate the appearance of symptomatology.

***Methodology*:** The kinetic in vitro study of the metabolism of histamine and putrescine was carried out in buffer (0.05 M and pH 7) supplemented with different concentrations of both amines and DAO enzyme of porcine origin. The solutions were kept at a constant temperature with regular stirring (37 °C, 200 rpm). Aliquots taken at 10 min intervals up to three hours were analyzed by ultra-high-pressure liquid chromatography with fluorescence detection (UHPLC-FL).

***Results and conclusions*:** It was found that putrescine was also a substrate of the DAO enzyme, although the rate of histamine metabolism was in all cases greater than the rate of putrescine, independent of the proportion of both amines in the solution. Individually, the reduction substrate at the 30 min test was 100% for histamine and approximately 50% for putrescine, which was not completely metabolized until the 120 min test. When the joint metabolism of both amines was considered, a decrease in the disappearance rate of histamine as the proportion of putrescine increased was observed. The results confirmed the hypothesis that putrescine from food reduces the metabolism rate of histamine by the DAO enzyme. These results may explain how high levels of putrescine in foods promote the intestinal absorption of histamine and its accumulation in plasma, which results in triggering symptoms of histamine intolerance. Therefore, in the dietary management of this intolerance, both histamine and putrescine contents should be considered.

#### 3.2.8. Probiotics with Diamine Oxidase Activity: Histamine Reduction Quantification and Location of the Responsible Gene

Comas-BastéO.Latorre-MoratallaM.L.RelatJ.BarcelóA.Veciana-NoguésM.T.Vidal-CarouM.C.

***Background and objectives*:** The use of probiotics with histaminase activity to help metabolize exogenous histamine in the intestine could be an alternative treatment for histamine intolerance by reducing DAO, thus broadening the spectrum of beneficial effects associated with probiotics. The ability to degrade histamine has been described in different microorganisms, mainly in bacteria used as starters. This activity has been shown as strain dependent and some authors have proposed that the gene coding for the histamine oxidase enzyme could be located in a plasmid. To our knowledge, this activity has not yet been described in any probiotic microorganism. The aim of this work was to study the histamine oxidase in vitro activity in *Lactobacillus gasseri* and *L. sakei* strains and the possible location of the gene that encodes this enzyme into a plasmid DNA.

***Methodology*:** Lactic acid bacteria have been identified with histaminase activity in vitro, specifically, *Lactobacillus sakei* and *L. gasseri* strains with reduction rates of 8%–50% and 70%–85%, respectively. The presence of plasmids was studied in four *L. sakei* strains and *L. gasseri* (1010 LU, LU 1011, 1015 LU, LU 1016) using a commercial kit (Miniprep plasmid PureYield™ System-A1220, Promega Biotech Ibérica, S.L., Madrid, Spain).

***Results and conclusions*:** No plasmid DNA was isolated from any of the tested strains, indicating that histamine oxidase activity is encoded in the genomic DNA. The fact that this gene was not located in plasmid DNA in the studied strains does not mean that it could not be located in a plasmid in other strains or species, so this study should be extended to other microorganisms. The responsible gene for histamine oxidase activity of *L. gasseri* was identified in genomic DNA by sequence alignment using the Basic Local Alignment Search Tool (BLAST), which allowed comparison between the results obtained from sequencing and sequences described in the literature for histamine oxidase activity in lactic bacteria.

#### 3.2.9. Effect of Composition of Tomato-Based Mediterranean *Sofrito* on Color Changes during Accelerated Storage

Cordero-GarcíaM.AlvarengaJ.F.R.Hurtado-BarrosoS.Lamuela-RaventósR.M.

***Background and objectives*:** Color in food is an important quality indicator of the degradation of product during storage and affects consumer-buying decision. The color of *sofrito* is related to the content of carotenoids, which are associated with health benefits. The objective of this study was to evaluate whether different ingredients of *sofrito* formulations might have an effect on color preservation during accelerated storage.

***Methodology*:** The effect of ingredients on the color change kinetics of *sofrito* was investigated during storage at 40 °C for 120 days. Eight formulations were made with the presence and absence of extra virgin olive oil (10%), onion (20%), garlic (2%) and the possible combinations between them. All samples were cooked for 30 min at 100 °C and packed in glass jars. Color changes during storage were measured by a colorimeter (model CR-410, Konica Minolta, Ramsey, NJ, USA). The brightness (L*), total color difference (∆E*), and hue (h°), as well as the kinetic models of zero, first and second order were evaluated by lineal regression.

***Results and conclusions*:** Results suggested that the presence of onion and extra virgin olive oil allowed a better preservation of color, as demonstrated by the hue values and the total color change kinetic model. Formulations containing garlic accelerated the degradation of color, indicated by good models of L* and suffered the most expressive total color change during storage. Thus, the presence of onion and extra virgin olive oil must be encouraged in the *sofrito* formulation to preserve the color and carotenoid content.

**Acknowledgments:** National Council for Scientific and Technological Development (CNPq, Brazil); The Ministry of Education, Culture and Sport (MECD); Spanish Ministry of Economy and Competitiveness (MEC); CIBER Physiopathology of Obesity and Nutrition (CIBEROBN), and Generalitat de Catalunya (Spain).

#### 3.2.10. Maternal Weight and Genetic Variants of the Fatty Acid Desaturase and Elongase Genes on Children’s Fatty Acids and Cognition

De la GarzaA.de AlmeidaL.ChisaguanoA.M.MontesR.BonillaM.DinarèsM.GuerendiainM.E.SalasI.CastelloteA.I.Torres-EspínolaF.J.Arias-GarcíaM.Segura-MorenoM.CampoyC.López-SabaterM.C.

***Background and objectives*:** Maternal polymorphisms (SNPs) in fatty acid desaturase (FADS) and elongase (ELOVL) genes alter long chain (LC) polyunsaturated fatty acid (PUFA) availability, compromising fetus supply and therefore cognitive development. Our aim was to determine how maternal polymorphisms in FADS and ELOVL genes influenced children’s fatty acids (FAs) and cognition according to maternal weight.

***Methodology*:** Children (*n* = 72) from the (Early Programming of Obesity) PREOBE cohort were divided according to maternal pre-pregnancy BMI: Group 1 (normoweight mothers, *n* = 31) and Group 2 (overweight/obese mothers, *n* = 41). Maternal SNPs were genotyped (seven in FADS1, five in FADS2, three in ELOVL2 and two in ELOVL5). At 18 months in age, children’s cheek cells were analyzed to measure PUFAs in the phospholipid fraction and cognition was assessed using the Bayley III Cognitive Scale.

***Results and conclusions*:** Major homozygotes in Group 1 had a higher arachidonic acid/dihomo-γ-linolenic acid index for rs174537 (FADS1) and higher cognition for rs174545 (FADS1) than minor allele carriers. Both tendencies persisted in all single-nucleotide polymorphisms (SNPs) in FADS1 while cognition tendency also persisted in FADS2. Group 2 showed that major homozygotes had higher cognition for rs2397142 (ELOVL5). Regarding rs2397142, Group 2 showed higher cognition when mothers carrying minor alleles had high docosahexaenoic acid intake and high plasma eicosapentaenoic acid/arachidonic acid and docosahexaenoic acid/arachidonic acid ratios.

Maternal weight, genotype and FAs influence children’s outcome. FADS1 SNPs in normoweight mothers decreased children’s cognition and enzymatic activity in FA metabolism. Children’s cognition was also lowered by ELOVL5 SNPs in obese, but not normoweight, mothers with low *n*-3 FA levels. A high *n*-3 FA intake should be promoted, especially in obese pregnancies, to enhance cognition in children.

#### 3.2.11. Effect of Adipokines Supplementation on Growth and Immunity in Suckling Rats

Grases-PintóB.Abril-GilM.Marín-MoroteL.Torres-CastroP.Rodríguez-LagunasM.J.CastellM.Pérez-CanoF.J.FranchÀ.

***Background and objectives*:** Breast milk contains bioactive factors that support the growth and development of the tissues and organ systems of newborns. Specifically, they promote the maturation of the digestive and immune systems and their physiological function. Among these bioactive factors, leptin and adiponectin—metabolic hormones or adipokines—may regulate physiological and metabolic processes, as well as the inflammatory response. However, the specific role of these adipokines in early life remains unexplored. The aim of this study was to establish the effects of leptin and adiponectin supplementation on rat growth and its development. It was also evaluated for its effect on the intestinal and systemic antibody response.

***Methodology*:** For this purpose, newborn Wistar rats were supplemented daily by oral gavage with leptin or adiponectin during the suckling period (21 days). In order to determine the morphometric variables, the body weight of the animals was assessed daily. At days 10, 14 and 21, the small intestine, spleen and thymus were obtained and weighed. Moreover, at day 21, plasma and gut washes were obtained to quantify systemic immunoglobulin A (IgA), immunoglobulin M (IgM), and immunoglobulin G (IgG) concentrations and intestinal IgA and IgM levels, respectively.

***Results and conclusions*:** Adipokines supplementation did not affect body growth. Leptin increased intestinal weight, whereas adiponectin increased thymus weight. Regarding the development of the immune system, leptin and adiponectin differentially modulated the intestinal and systemic antibody response in early life.

**Acknowledgments:** This study was supported by the Spanish Ministry of Economy and Competitiveness (AGL2013-48459-P).

#### 3.2.12. Bioavailability of Carotenoids *Sofrito* in Healthy and Young Men Following a Diet Rich in Foods with Antioxidant Compounds

Hurtado-BarrosoS.Martínez-HuélamoM.Colmán-MartínezM.AlvarengaJ.F.R.Lamuela-RaventósR.M.

***Background and objectives*:** The *sofrito* is a typical sauce in a Mediterranean diet that contains tomato as the main component and other plant-based ingredients (olive oil, onion and garlic) rich in bioactive compounds such as carotenoids. However, bioavailability is low and various factors such as processing and food matrix may interfere positively and/or negatively. The aim of this study was to evaluate the bioavailability of carotenoids in *sofrito* after a diet rich in antioxidants.

***Methodology*:** A pilot study was performed on twenty-two healthy, non-smoking men, aged between 18 and 32 years who consumed *sofrito* (at a dose of 240 g/70 kg). Identification and quantification of carotenoids in the plasma collected at 0, 5 and 24 h was carried out by high-pressure liquid chromatography with ultraviolet detector (HPLC-UV), 1100HPLC HP system (Hewlett-Packard, Waldbronn, Germany).

***Results and conclusions*:** Carotenoids increased significantly (*p* > 0.05) between zero and 24 h (3.227 ± 1.738 vs. 7.630 ± 3.933) and between five and 24 h (4.570 ± 2.524 vs. 7.630 ± 3.933). Specifically, lycopene increased 24 h after sauce intake (2.731 ± 1.089 vs. 5.816 ± 3.332). However, no significant differences were found among xanthophylls (0.624 ± 0.083 vs. 0.603 ± 0.221 vs. 1.010 ± 0.838) after intervention. The carotenoids in plasma increased significantly after *sofrito* intake, mainly lycopene after 24 h of *sofrito* intervention.

**Acknowledgments:** This study was funded by the Ministry of Economy and Competitiveness (AGL2013-49083-C3-1-R) and CIBER Physiopathology of Obesity and Nutrition (CIBEROBN).

#### 3.2.13. Tyramine and Histamine Risk Assessment Related to Consumption of Dry Fermented Sausages by the Spanish Population

Latorre-MoratallaM.L.Comas-BastéO.Sánchez-PérezS.Bover-CidS.Vidal-CarouM.C.

***Background and objectives*:** Tyramine and histamine are bioactive amines involved in the appearance of adverse health effects in at risk population subgroups. A high intake of tyramine can trigger hypertensive disorders, among others, especially when combined with the administration of drugs that inhibit the metabolism of this amine (monoamine oxidase inhibitors (MAOI) or reversible inhibitors of monoamine oxidase A (RIMA)). In the case of histamine, intoxication symptoms may occur due to an excessive intake of this amine or as a result of the intolerance caused by blockage or deficit diamino oxidase (the enzyme responsible of the intestinal metabolism of histamine). Dry fermented sausages, which are extensively consumed in Spain, can easily accumulate high levels of these hazards. The aim of this study was to evaluate the extent that the consumption of dry, fermented sausages could contribute to the appearance of these adverse health effects on an at-risk Spanish population.

***Methodology*:** Probabilistic estimation of tyramine and histamine intake was performed using the Monte Carlo simulation technique (@Risk 7.0), by combining the distributions of the content of these amines in dry fermented sausages (*n* = 474) with the consumption data of the Spanish population (ENIDE, “Encuesta Nacional de Ingesta Dietética Española”). For the risk assessment, the maximum tolerable safety levels adopted by European Food Safety Authority (EFSA); the percentage of potential meat product consumers in Spain; data on MAOI drug usage (Spanish Agency of Medicines and Sanitary Products); and the prevalence of histamine intolerance were considered.

***Results and conclusions*:** The risk of suffering hypertensive crisis or histamine intoxication through the consumption of dry, fermented sausages in a healthy population may be considered negligible. Conversely, the results confirmed that consumption of fermented sausages carried risk in patients treated with MAOI drugs, as well as in people diagnosed with histamine intolerance. In particular, individuals undergoing treatment with MAOI drugs have a high risk of hypertensive crisis, with a probability of 34% exceeding the safety threshold of tyramine (six milligrams) in a meal. However, given the reduced actual usage of these drugs, only three out of a million people could suffer these adverse effects. With regard to histamine intolerance, EFSA has not established a safe limit for histamine since the risk of the onset of symptoms varies depending on the degree of DAO deficit. 66% of sausages contained variable amounts of histamine (<0.01 to 475 mg/kg), therefore their consumption is liable to trigger symptoms. Considering that approximately 1% of the population is histamine intolerant, the population at risk would reach 7000 cases per million individuals.

#### 3.2.14. Serum Diamine Oxidase (DAO) Activity Levels in Patients with Migraine

Latorre-MoratallaM.L.Comas-BastéO.Izquierdo-CasasJ.Soler-SinglaL.Lorente-GascónM.DueloA.Vidal-CarouM.C.

***Background and objectives*:** Histamine intolerance is a disorder in the homeostasis of histamine due to a reduced enzymatic intestinal DAO activity, which causes an accumulation of this amine in plasma. Some authors have associated DAO deficiency with several pathologies of high prevalence in the population, such as migraine, inflammatory and degenerative intestinal disorders or atopic dermatitis. A better knowledge of serum DAO levels in the migranous population is important in order to establish the relationship between histamine intolerance by DAO deficit and migraine. The objective of this study was to determine the prevalence of DAO deficiency in healthy subjects and in patients with a confirmed migraine diagnosis.

***Methodology***: The prevalence of DAO deficiency was assessed in a total of 198 volunteers recruited at the Headache Unit of the Hospital General de Catalunya; 137 had a confirmed migraine diagnosis (according to current International Headache Society criteria) and 61 healthy volunteers without clinical criteria for migraine considered as a control group. Blood samples were collected from all subjects by venipuncture with an ethylenediaminetetraacetic acid tube after an eight-hour fasting period and the samples were analyzed with enzyme-linked immunosorbent assay to determine DAO enzyme activity. Values below 80 HDU/mL (Histamine Degradation Units/mL) were considered as DAO deficient.

***Results and conclusions*:** The mean value of DAO activity of the migraine population (64.5 ± 33.5 HDU/mL) was significantly lower (*p* < 0.0001) than that obtained from healthy volunteers (91.9 ± 44.3 HDU/mL). A high incidence rate of DAO deficiency (88%) was observed in the group of patients with migraine. In addition, 44% of non migranous subjects had levels of DAO activity lower than 80 HDU/mL, and no information about other pathologies associated with DAO deficiency was registered. DAO deficiency was more prevalent in migraine patients than in the healthy population. Further studies are needed in order to test whether DAO deficiency is the cause of triggering migraine. In that case, a free histamine diet and/or DAO supplementation could play an important role in the treatment of this disorder.

#### 3.2.15. Nutrimetabolomic Approach to Identify Biomarkers for Cocoa-Food Products Consumption Monitoring in Healthy Volunteers

LlorachR.Farran-CodinaA.SorianoA.Termes-EscaléM.LunaO.MartinezC.BoschN.GarridoP.LlobetJ.M.Andres-LacuevaC.Urpi-SardaM.

***Background and objectives*:** Cocoa consumption has been linked to health-promoting activities mainly associated with cardiovascular disease. Nutrimetabolomics explores the complex relationship between the consumption of dietary compounds and the maintenance of health or disease development, with the aim to discover new biomarkers of intake and effect. In this regard, the aim of this work was to study the urinary cocoa product fingerprint for future definition of a specific biomarker imprint of cocoa in healthy and young volunteers.

***Methodology*:** The cocoa-food products intake was defined according to a food frequency questionnaire, which was previously completed by the free-living healthy volunteers. Subjects were classified as high (≥5 g/day), medium (between 1.16 and 4.28 g/day), and low (≤1 g/day) consumers. Urine samples from the subjects were analyzed by high-pressure liquid chromatography—time of flight mass spectrometry (HPLC-Q-TOF-MS), followed by multivariate data analysis (orthogonal signal correction for partial least squares discriminant analysis (OSC-PLSDA) and hierarchical cluster analysis (HCA)). The metabolomics analysis was carried out using a R package “Metabolite Automatic Identification Toolkit” (MAIT) that included an in-house food metabolome database.

***Results and conclusions*:** Urinary metabolome showed significant differences between the three consumer groups. Several metabolites were associated with cocoa-foods consumption, the most important derived from theobromine metabolism. In addition, microbial polyphenol metabolites and vanillin-derived metabolites were putatively identified. These metabolites have previously been associated with cocoa intake in other populations, however, as far we know, this is the first time that these metabolites have been identified in a free-living, healthy and young population using a metabolomics approach. This study reinforced interest in the replication of results, as well as the capacity of metabolomics to identify the cocoa-food product footprint by combining epidemiological nutritional data and metabolomics.

**Acknowledgments:** This work was supported by a FRI-13 award from INSA-UB (2014–2015). We also thank the EU Joint Programming Initiative A Healthy Diet for a Healthy Life on Biomarkers BioNHFOODBALL (PCIN-2014-133—Spanish Ministry of Economy and Competitiveness (MINECO)) and the award of 2014SGR1566 from the Generalitat de Catalunya’s Agency AGAUR. Mireia Urpi-Sarda would like to thank the “Ramón y Cajal” program from MINECO and the Fondo Social Europeo.

#### 3.2.16. Food Metabolome Biomarkers Associated with Dietary Patterns in Healthy Volunteers

LlorachR.Farran-CodinaA.SorianoA.Termes-EscaléM.LunaO.MartinezC.BoschN.GarridoP.LlobetJ.M.Andres-LacuevaC.Urpi-SardaM.

***Background and objectives*:** There is a growing interest in the field of nutrition epidemiology to identify biological markers related to the intake of nutrients, foods and dietary patterns associated with the prevention of diseases. The aim of this study was to identify urinary biomarkers to describe the dietary patterns in healthy young individuals.

***Methodology*:** This was an observational study undertaken at the Campus de l’Alimentació-Torribera (University of Barcelona) during the academic year of 2014–2015. Forty-four healthy volunteers were included in the study, based on the following criteria: non-smoking, healthy and with an age between 18 and 25 years old. Participants recorded their dietary habits with a validated food frequency questionnaire (FFQ). Participants collected 24 h urine samples in two different visits (separated by three months). FFQ data were grouped into 28 categories and a k-means cluster analysis (Metaboanalyst 3.0) was used to identify the dietary patterns. Urine was analyzed by high-pressure liquid chromatography—time of flight mass spectrometry (HPLC-q-Tof) (Applied Biosystems). Differences in the urinary biomarkers between patterns were analyzed using the metabolomics R package MAIT.

***Results and conclusions*:** K-means analysis identified two dietary patterns; one characterized by a significant higher intake of whole grain cereals, fruit, vegetables, pulses, coffee, moderate wine, nuts and dried fruit (*p* < 0.05) (High Dietary Vegetables Pattern, HDP); while the other cluster was characterized by a significant higher intake of refined grain cereals, processed meats, snacks and high energy beverages (*p* < 0.05) (Low Dietary Vegetable Pattern, LDP). Urinary food metabolome showed differences between both dietary patterns. The HDP was characterized by a higher urinary excretion of urolithin A glucuronide, a characteristic microbial metabolite of nut consumption; of 4-hydroxyhippuric acid, a microbial metabolite related to higher intake of vegetables and derived foods; proline betaine, which is related to orange consumption; and trigonelline, a metabolite linked to coffee consumption. The LDP was characterized by a higher urinary excretion of tyrosine sulfate, an endogenous metabolite associated to cardiovascular risk factors. In conclusion, these results indicated that HDP and LDP are reflected in the urinary metabolome. Among the HDP biomarkers, it is important to note that microbial derived metabolites could be very useful in evaluating food intake in epidemiological studies. Furthermore, LDP biomarkers could be linked to future diseases in epidemiological studies.

**Acknowledgments:** This work was supported by a FRI-13 award from INSA-UB (2014–2015). We also thank the EU Joint Programming Initiative A Healthy Diet for a Healthy Life on Biomarkers BioNHFOODBALL (PCIN-2014-133-MINECO-Spain) and the award of 2014SGR1566 from the Generalitat de Catalunya’s Agency AGAUR. Mireia Urpi-Sarda would like to thank the “Ramón y Cajal” program from MINECO and the Fondo Social Europeo.

#### 3.2.17. Development of an Advanced High Pressure Liquid Chromatography Tandem Mass Spectrometry (HPLC-MS/MS) Method for the Determination of Carotenoids and Fat-Soluble Vitamins in Human Plasma

Martínez-HuélamoM.HrvolováB.Colmán-MartínezM.Hurtado-BarrosoS.KalinaJ.Lamuela-RaventósR.M.

***Background and objectives*:** Although carotenoids and fat-soluble vitamins are of utmost interest in human health, sensitive and specific methods for their simultaneous determination are scarce. The aim of this research was to develop and validate a new high-pressure liquid chromatography tandem mass spectrometry (HPLC-MS/MS) method for the quantification of selected carotenoids and fat-soluble vitamins in human plasma.

***Methodology*:** The samples were extracted using a double liquid-liquid extraction method with *n*-hexane/butylated hydroxytoluene (100 mg/L). HPLC-MS/MS was carried out in an Agilent 1100 HPLC system coupled to a QTRAP4000 triple quadrupole mass spectrometer.

***Results and conclusions*:** In 50 min, 16 samples were separated with an excellent resolution and suitable mass signal intensity. The proposed HPLC-MS/MS method led to improvements in the limits of detection (0.001 to 0.422 μg/mL) and quantification (0.003 to 1.406 μg/mL) for all 16 analyzed compounds when compared with the most often used HPLC-DAD methods, in some cases being more than 100-fold lower. The recovery (86.1%–104.8%), accuracy (85.9%–114.0%), and precision (<14%) met with the acceptance criteria of the Association of Official Analytical Chemists International.

According to these results, the described HPLC-MS/MS method was adequately sensitive, repeatable and suitable for the large-scale analysis of compounds in biological fluids.

**Acknowledgments:** This work was supported by the Interagency Council on Science and Technology (CICYT) (AGL2013-49083-C3-1-R); the Instituto de Salud Carlos III (ISCIII), CIBER Physiopathology of Obesity and Nutrition (CIBEROBN) from the Spanish Ministry of Economy and Competivity (MEC); Generalitat de Catalunya (GC) 2014 SGR 773; and by the Project LO1208 (TEWEP) of the National Feasibility Programme I of the Czech Republic. Hrvolová, B. also thanks the student grant n. SGS04/PřF/2016 from the University of Ostrava, Czech Republic.

#### 3.2.18. Organoleptic Characteristics of Quality Spanish Sparkling Wine “Cava”

MirandaI.Riu-AumatellM.BuxaderasS.López-TamamesE.

***Introduction and objectives*:** During ageing, the enologists describe the evolution of the sensorial characteristics of cava from fruity (due to acetate and ethyl esters) to more complex sensations as nuts, warm, and mature fruits due to the particular ageing from contact with lees (non-viable cells of *Saccharomyces cerevisiae*).

The characterization of the aroma of Spanish sparkling wine is useful in order to find strategies to improve cava volatile composition. Furthermore, 200 tons of lees were produced every year and are considered as a by-product of the enology industry. With the aim to exploit this by-product, cava was enriched with the volatiles encapsulated in lees in order to study their behavior in the sparkling wine.

***Methodology*:** Volatile composition was obtained by Headspace–Solid Phase Microextraction (HS-SPME) coupled to gas chromatography mass spectrometry (GC/MS). The encapsulation was produced by diffusion.

***Results and conclusions*:** As expected, the aroma of cava was composed of numerous chemical families such as esters, alcohols, norisoprenoids, terpenes, and acids. In order to improve the organoleptic quality of cava, the wine was enriched with two volatiles encapsulated with yeast lees of second fermentation (*S. cerevisiae*). The release of the volatiles occurred in only 24 h. Thus, the reutilization of this enology industry by-product was proposed in order to increase the organoleptic characteristics of wine.

**Acknowledgments:** Freixenet S.A. for providing samples. The Interagency Council on Science and Technology (CICYT) projects AGL2011-23872 and AGL2016-78324-R.

#### 3.2.19. Combined Determination of Polyphenols and Pentacyclic Triterpenes from Table Olives by High Pressure Liquid Chromatography Mass Spectrometry (HPLC-MS)

Moreno-GonzálezR.KundisovaI.Gómez-ContrerasA.EkşiH.JuanM.E.PlanasJ.M.

***Background and objectives*:** Table olives are typical components of the Mediterranean Diet that could contribute to its beneficial health effects given its high content of oleic acid, as well as other bioactive compounds. With the aim of increasing knowledge on the composition of pentacyclic triterpenes and polyphenols in olives, a method that enables the simultaneous extraction of both groups of compounds has been developed.

***Methodology*:** Polyphenols (hydroxytirosol, tyrosol, and oleuropein) and pentacyclic triterpenes (maslinic acid, oleanolic acid, and erythrodiol) were simultaneously extracted from arbequina table olives using ethanol–methanol (1:1 *v*/*v*) as a solvent, which includes a different internal standard for triterpenes and polyphenols. After three consecutive extractions that included vigorous vortexmixing and centrifugations, the supernatants were analyzed by high-pressure liquid chromatography atmospheric pressure chemical ionization mass spectrometry (HPLC-APCI-MS) to determine pentacyclic triterpenes and by HPLC-electrospray ionization mass spectrometry for polyphenols.

***Results and conclusions*:** The validation results showed that the method was linear (*r* > 0.99), sensitive, precise (CV < 15%) and accurate for all analyses, with a recovery >88% for triterpenes and >94% for polyphenols. The analysis of Arbequina olives indicated that maslinic acid was the main bioactive compound (2585.6 ± 90.8 mg/kg) followed by oleanolic acid (741.6 ± 13.5 mg/kg), hydroxytirosol (458.7 ± 88.1 mg/kg) and tirosol (41.7 ± 9.04 mg/kg), with minor amounts of erythrodiol (9.48 ± 0.34 mg/kg) and oleuropein (1.38 ± 0.49 mg/kg). The development of this method provides a reliable and accurate combined extraction of chemically different compound, thus allowing a rapid analysis of polyphenols and pentacyclic triterpenes in olives.

**Acknowledgments:** Supported by AGL2013-41188 (Spanish Ministry of Economy and Competitiveness) and 2014SGR1221 (Generalitat de Catalunya).

#### 3.2.20. Dietary Spray-Dried Plasma (SDP) Supplementation Promotes Anti-Inflammatory Mediators in Mice Challenged with *S. aureus* Enterotoxin B

MoretóM.MiróL.PoloJ.AmatC.Pérez-BosqueA.

***Background and objectives*:** In rodents, dietary supplementation with spray-dried plasma (SDP) attenuates lymphocyte activation in mesenteric lymph nodes (MLN) during intestinal inflammation induced by *S. aureus* enterotoxin B (SEB). The objective of this study was to discern the molecular mechanisms involved in the intestinal protective effects of SDP.

***Methodology*:** Male C57BL/6 mice were fed with 8% SDP or milk proteins for two weeks, from weaning until day 33. On day 32, mice were given a single SEB dose (i.p., 25 µg/mice) or phosphate buffered saline (PBS). Twenty-four hours later, mice were sacrificed and MLN lymphocytes were stained and analyzed by flow cytometry. The expression of cytokines, adhesion molecules and transcription factors were determined in the intestinal mucosa.

***Results and conclusions*:** SEB increased the MLN cell recruitment and the percentage of activated Th lymphocytes (*p* < 0.05), which were attenuated by SDP (*p* < 0.05). The enterotoxin did not change the mucosal expression of IL-10 and TGFβ1, but SDP increased the expression of both cytokines when compared to the SEB group (*p* < 0.05). The enterotoxin increased the expression of Madcam-1 and ICAM-1 (*p* < 0.001) and these effects were attenuated by SDP (*p* < 0.05). SEB also decreased Smad2/3 phosphorylation and augmented NFκB phosphorylation (*p* < 0.05), which were prevented by SDP (*p* < 0.05). Our results indicated that SDP modulates the immune response in challenged animals through the regulation of transcription factors and adhesion molecules that, in turn, will reduce intestinal cell infiltration and the magnitude of the inflammatory response.

**Acknowledgments:** Supported by 2014SGR1221 (Generalitat de Catalunya, Spain) and Fundació Bosch i Gimpera (UB) FBG 306994 contract.

#### 3.2.21. Urine Metabolome Changes after Beer and Non-Alcoholic Beer Intake

Quifer-RadaP.Chiva-BlanchG.JaureguiO.EstruchR.Lamuela-RaventosR.

***Background and objectives*:** Moderate alcohol consumption is associated with a decrease in cardiovascular risk, but fermented beverages seem to confer greater cardiovascular protection due to their polyphenolic content. Beer contains a wide range of polyphenols derived from the malt or hops used during brewing, including simple phenolic acids, hydroxycinnamoylquinics, flavanols, flavonols, flavones, alkylmethoxyphenols, alpha- and iso-alpha-acids, hydroxyphenylacetic acids, and prenylflavanoids.

The objective of this work was to compare the effects of moderate consumption of beer, non-alcoholic beer and gin on the overall urine metabolome.

***Methodology*:** A crossover trial with 33 men at high cardiovascular risk was randomized to receive beer (30 g alcohol/day), the equivalent amount of polyphenols in the form of non-alcoholic beer, or gin (30 g alcohol/day) for four weeks. Urine samples were analyzed by liquid chromatography–high-resolution mass spectrometry (HPLC-MS/MS). A combination of univariate statistical analysis, multivariate analysis (partial least squares discriminant analysis), data-dependent MS/MS scan, and accurate mass database matching was used to measure the effect of beer, non-alcoholic beer and gin intake in the urinary metabolome.

***Results and conclusions*:** A total of 10 metabolites were identified. Eight were exogenous metabolites related to beer, non-alcoholic beer or gin consumption, but two of them were related to metabolic endogenic changes: hydroxyadipic acid linked to fatty acid oxidation, and 4-guanidinobutanoicacid involved in arginine metabolism. Humulinone, a hop bitter acid normally found in beer, could be a novel biomarker of beer and nonalcoholic beer consumption.

**Acknowledgments:** This study was supported by The European Foundation for Alcohol Research (ERAB) EA 1117 and EA 1324 and CIBER Physiopathology of Obesity and Nutrition (CIBEROBN).

#### 3.2.22. Are the Olive Oil Sesquiterpenes Influenced by Hydric Stress?

Quintanilla-CasasB.VichiS.De CariaS.PrietoM.H.LaraE.Pérez-RodríguezJ.M.

***Background and objectives*:** The virgin olive oil (VOO) market has evolved in the last few years with the appearance of quality labels as denominations and protected indications of origin. For this reason, it is necessary to find analytical traceability markers to avoid fraudulent claims made to consumers. Volatile compounds could be used for this purpose, especially the products of the lipoxygenase pathway, but most of them are affected by several technological parameters during olive processing which reduces their usefulness. Nevertheless, semi-volatile compounds from the secondary metabolism of olive fruit are also found in the oil: the sesquiterpene hydrocarbons, which are not significantly modified by the oil extraction process, could be a suitable tool for the geographical and varietal authentication of VOO. To corroborate the usefulness of sesquiterpenes as traceability markers, it was necessary to evaluate their dependence on agronomic factors that could affect VOO sesquiterpene composition. The evaluation of irrigation, as one of the essential agronomic factors was the aim of the present study.

***Methodology*:** Sesquiterpene composition was evaluated by solid phase microextraction–gas chromatography (SPME-GC/MS) in VOO samples from three consecutive crop years (2009–2011) that were obtained from the same olive cultivar (Arbequina) and region and subjected to four different irrigation treatments.

***Results and conclusions*:** Sesquiterpene distribution in VOO was influenced by markedly different water availability and depended on their structure. Cyclic sesquiterpenes were proportionally related to hydric stress while linear sesquiterpenes and some terpene ketones are favored by water availability.

#### 3.2.23. Polyphenol Exposure Is Inversely Associated with Geriatric Conditions, Frailty and Mortality within the Invecchiare in Chianti (InCHIANTI) Cohort: Relevance to Polyphenol Recommendations

RabassaM.Zamora-RosR.CherubiniA.Urpi-SardaM.BandinelliS.FerrucciL.Andres-LacuevaC.

***Background and objectives*:** There is growing evidence on the health-protective role of dietary polyphenols intake on aging, but the assessment of dietary polyphenols intake from self-reported questionnaires tends to be inaccurate and unreliable. A promising alternative is the use of urinary polyphenols concentration as a more accurate measure of intake. The aim was to investigate the association between dietary polyphenols and urinary polyphenol concentrations; and cognitive and physical decline, frailty phenotype, and all-cause mortality among older adults aged ≥65 years or more within the Invecchiare in Chianti (InCHIANTI) study, an Italian cohort.

***Methodology*:** The dietary intake of total polyphenols and resveratrol was estimated using a validated food frequency questionnaire and an ad hoc database of polyphenols in food composition. The urinary concentration of total polyphenols and resveratrol was determined using the Folin–Ciocalteu colorimetric and mass spectrometry methods, as biomarkers of the dietary total of polyphenols and resveratrol, respectively.

***Results and conclusions*:** The highest tertile of total urinary polyphenols was inversely associated with the risk of cognitive and physical decline, frailty and total mortality, in comparison with the lowest tertile. However, no association with total dietary polyphenols was observed. Additionally, habitual dietary exposure of resveratrol was associated with a lower risk of developing frailty in a combination of both measures (diet and biomarker), as well as individually. In conclusion, these results suggested the protective effect of a polyphenol-rich diet against cognitive and physical decline, frailty and all-cause mortality in older persons. Furthermore, it demonstrates the importance of assessing dietary polyphenol exposure, whenever possible, using nutritional biomarkers and not only self-reported questionnaires.

**Acknowledgments:** This research was supported by the Consolider Ingenio 2010 Program Acronym: FUN-C-FOOD (CSD2007-063); the International Nut and Dried Fruit Council Foundation (INC) (FBG307906); and the Joint Programming Initiative—A Healthy Diet for a Healthy Life (JPI HDHL) Acronym: FOODBALL (Ministry of Economy and Competitiveness (PCIN-2014-133).

#### 3.2.24. Norovirus Shedding among Food and Healthcare Workers Exposed to the Virus in Outbreak Settings

SabriàA.PintóR.M.BoschA.BartoloméR.CornejoT.TornerN.MartínezA.de SimónM.DomínguezA.GuixS.

***Background and objectives***: Noroviruses (NoVs) are the leading cause of nonbacterial outbreaks of gastroenteritis worldwide. Individuals who are asymptomatically infected may facilitate the transmission of NoVs. Our aim was to evaluate the occurrence of NoVs infections among workers exposed to the virus in different outbreak settings.

***Methodology*:** We screened feces from food handlers and healthcare workers related to gastroenteritis outbreaks, and shedding concentrations over time were calculated from serial samples of infected individuals. Sequence analyses of the capsid P2 domain and region C were used to evaluate the link between asymptomatic employees and outbreak cases.

***Results and conclusions***: Of all employees, 59.1% were positive for NoVs, and more than 70% of them were asymptomatic. Asymptomatic infections were significantly more frequent among food handlers compared to healthcare workers. Mean viral loads were similar between symptomatic and asymptomatic individuals, starting at 7.51 ± 1.80 and 6.49 ± 1.93 log_10_ genome copies/g, respectively, and decreasing to 5.28 ± 0.76 and 4.52 ± 1.45 log_10_ genome copies/g after 19 days.

In the setting of a NoVs outbreak, workers show a high risk of becoming infected. Since shed amounts of viruses without symptoms are also high, reinforcement of hygiene practices among workers is relevant to reduce the risk of virus secondary transmissions.

**Acknowledgments:** Thanks to the Working Group for the Study of Outbreaks of Acute Gastroenteritis in Catalonia; the physicians who reported outbreaks; and the Epidemiological Surveillance Units of the Department of Health of the Government of Catalonia and the Public Health Agency of Barcelona.

#### 3.2.25. Effects of Different Doses of Polyphenols from Dealcoholized Red Wine on Endothelial Function in Subjects with Metabolic Syndrome and High Cardiovascular Risk

SasotG.Creus-CuadrosA.Mercader-MartíM.Lamuela-RaventósR.M.EstruchR.

***Background and objectives*:** Several studies have pointed out that mortality and risk from cardiovascular disease (CVD) are higher in subjects with Metabolic Syndrome (MetS), which is considered as a cluster of risk factors. Epidemiological studies and intervention clinical trials have shown that dealcoholized red wine (DRW) and moderate consumption of red wine (RW) are inversely associated with cardiovascular risk factors.

***Methodology*:** A randomized, open prospective trial, running in parallel with a controlled clinical trial, was performed with 54 subjects with MetS to evaluate the effects of polyphenols. Doses of 375 mL/day of DRW, dealcoholized red wine with grape extract (DRWEx) or water were administered during three months. Anthropometric measurements and blood biochemical analysis were carried out at the baseline and after each intervention. Levels of endothelial progenitor cells (EPC), circulating endothelial cells (CEC) and leukocyte cell membrane receptors were analyzed by flow cytometry.

***Results and conclusions*:** After DRW and DRWEx interventions, we observed a decrease in body weight, body mass index, waist circumference, total cholesterol concentration, and an improvement on blood pressure. Moreover, there was an increase in the number of EPCs and a significant decrease in levels of CECs and T-lymphocyte expression. We concluded that the non-alcoholic fraction of wine, rich in polyphenols, may reduce cell adhesion molecules and CEC, known markers of CVD severity and increase EPC, a marker of endothelial regeneration, in a population at high risk of CVD due to MetS. These features may explain why DRW and moderate RW consumption suggest an improvement in the condition of the vascular endothelium and possibly contributes in delaying the development of atherosclerotic plaques.

**Acknowledgments:** Authors would like to express their gratitude for financial support from the INCOMES project supported by the Spanish Ministry of Economy and Competitiveness through the INNPRONTA program, as well as the Interagency Council on Science and Technology (CICYT) (AGL2013-49083-C3-1-R); Generalitat de Catalunya FI-DGR 2013/FI-DGR 2014; the Instituto de Salud Carlos III (ISCIII) and CIBER Physiopathology of Obesity and Nutrition (CIBEROBN) from the Spanish Ministry of Science and Innovation (MICINN).

#### 3.2.26. Urine Polyphenol Excretion Is Inversely Correlated with Body Weight in the “Prevención con Dieta Mediterránea” (PREDIMED) Population

Tresserra-RimbauA.GuoX.EstruchR.Medina-RemónA.Martínez-GonzálezM.A.FitóM.CorellaD.Salas-SalvadóJ.Lamuela-RaventósR.M.on behalf of the PREDIMED Study Investigators

***Background and objectives*:** The high prevalence of overweight and obesity is one of the most common causes of morbidity and mortality. Although polyphenol intake has been associated with their protection against several chronic diseases, only a few human studies have investigated the role of polyphenols in body weight. Our aim was to investigate whether urinary polyphenol levels are associated with obesity parameters.

***Methodology*:** A longitudinal study was performed with 573 participants from the PREDIMED (Prevención con Dieta Mediterránea) trial (ISRCTN35739639). Total polyphenol excretion (TPE), as a biomarker of polyphenol intake, was determined by the Folin–Ciocalteu method in urine samples. Participants were categorized into five groups according to TPE at five years. Multiple linear regression models were used to assess the relationships between TPE and obesity parameters.

***Results and conclusions*:** After a five-year follow-up, significant inverse correlations were observed between five-year TPE and body weight; body mass index; waist circumference; and waist-to-height ratio, after adjustments for potential confounders. Compared with those in the lowest quintile, participants in the top TPE quintile showed a lower prevalence of obesity.

A greater polyphenol intake may therefore contribute to the reduced risk of obesity in elderly people who are at high cardiovascular risk.

**Acknowledgments:** This study was supported by the Interagency Council on Science and Technology (CICYT) (AGL2016-75329-R) from the Spanish Ministry of Economy and Competitiveness (MEC); the Generalitat de Catalunya (GC) 2014 SGR 773; and the Instituto de Salud Carlos III (ISCIII) and CIBER Physiopathology of Obesity and Nutrition (CIBEROBN). CIBEROBN is an initiative of ISCIII, Spain. X. Guo received support from China Scholarship Council (CSC). A. Medina-Remón thanks the “Juan de la Cierva” postdoctoral program (JCI-2012-13463) from MEC.

## Appendix A. Participant Author’s Affiliations

- Abril-Gil, M. Section of Physiology, Department of Biochemistry and Physiology, Faculty of Pharmacy and Food Sciences, University of Barcelona, Barcelona, Spain; Nutrition and Food Safety Research Institute (INSA-UB), Santa Coloma de Gramenet, Spain.
- Alda, J.A. ADHD Unit. Child and Adolescent Psychiatry Department. Hospital Sant Joan de Deu, Barcelona, Spain.
- Almanza-Aguilera, E. Biomarkers and Nutrimetabolomic Lab, Department of Nutrition, Food Sciences and Gastronomy, XaRTA, INSA, Food and Nutrition Torribera Campus, Faculty of Pharmacy and Food Science, University of Barcelona, Santa Coloma de Gramenet, Spain; CIBER Frailty and Healthy Aging (CIBERFES), Institute of Health Carlos III, Madrid, Spain.
- Alvarenga, J.F.R. Nutrition, Food Science and Gastronomy Department, XaRTA, INSA, Pharmacy and Food Science School, University of Barcelona, Barcelona, Spain.
- Amat, C. Section of Physiology, Department of Biochemistry and Physiology, Faculty of Pharmacy and Food Sciences; Nutrition and Food Safety Research Institute, University of Barcelona (UB), Spain.
- Andres-Lacueva, C. Biomarkers and Nutrimetabolomic Lab, Department of Nutrition, Food Sciences and Gastronomy, XaRTA, INSA, Food and Nutrition Torribera Campus, Faculty of Pharmacy and Food Science, University of Barcelona, Santa Coloma de Gramenet, Spain; CIBER Frailty and Healthy Aging (CIBERFES), Institute of Health Carlos III, Madrid, Spain.
- Arias García. M. Centre of Excellence for Paediatric Research EURISTIKOS, University of Granada, Granada, Spain.
- Arranz-Martínez, S. Department of Internal Medicine, Hospital Clinic, IDIBAPS, University of Barcelona; CIBER Physiopathology of Obesity and Nutrition (CIBEROBN), Health Institute “Carlos III”, Madrid, Spain.
- Arteaga, C. Toxicology Unit, Department of Pharmacology, Toxicology and Medicinal Chemistry, Faculty of Pharmacy and Food Sciences, University of Barcelona, Barcelona, Spain.
- Azagra-Boronat, I. Section of Physiology, Department of Biochemistry and Physiology, Faculty of Pharmacy and Food Sciences, University of Barcelona, Barcelona, Spain; Institute for Research on Nutrition and Food Safety (INSA-UB), Barcelona, Spain.
- Bandinelli, S. Geriatric Rehabilitation Unit, Azienda Sanitaria Firenze, Florence, Italy.
- Barceló, A. Service Genomics and Bioinformatics, Institute of Biotechnology and Biomedicine (IBB), Research Park, Autonomous University of Barcelona, Bellaterra, Spain.
- Bartolomé, R. Laboratory of Microbiology, Hospital Universitari Vall d’Hebron, Barcelona, Spain.
- Bayés-García, L. Section of Crystallography, Mineralogy and Mineral Deposits, Faculty of Earth Sciences, University of Barcelona, Bellaterra, Spain.
- Boix, N. Toxicology Unit, Department of Pharmacology, Toxicology and Medicinal Chemistry, Faculty of Pharmacy and Food Sciences, University of Barcelona, Bellaterra, Spain.
- Bonilla, M. Department of Nutrition, Food Science and Gastronomy-XARTA-INSA, School of Pharmacy and Food Science, University of Barcelona, Barcelona, Spain.
- Bosch, A. Enteric Virus Group, Department of Microbiology, University of Barcelona, Barcelona, Spain; Nutrition and Food Safety Research Institute (INSA-UB), University of Barcelona, Santa Coloma de Gramanet, Spain.
- Bosch, N. Department of Nutrition, Food Sciences and Gastronomy, Food and Nutrition Torribera Campus, Faculty of Pharmacy and Food Science, University of Barcelona, Santa Coloma de Gramenet, Spain.
- Bover-Cid, S. IRTA-Food Safety Programme, Institute for Food and Agricultural Research and Technology, Monells (Girona), Spain.
- Bravo Clemente, L. Institute of Food Science and Technology and Nutrition (ICTAN-CSIC).
- Buxaderas, S. Department of Nutrition, Food Sciences and Gastronomy, XaRTA, INSA, Food and Nutrition Torribera Campus, Faculty of Pharmacy and Food Science, University of Barcelona, Santa Coloma de Gramenet, Spain.
- Calvet, T. Section of Crystallography, Mineralogy and Mineral Deposits, Faculty of Earth Sciences, University of Barcelona, Bellaterra, Spain.
- Campoy, C. University of Granada, Centre of Excellence for Paediatric Research EURISTIKOS, Granada, Spain; University of Granada, Paediatrics, Granada, Spain.
- Camps-Bossacoma, M.Section of Physiology, Department of Biochemistry and Physiology, Faculty of Pharmacy and Food Sciences, University of Barcelona, Barcelona, Spain; Nutrition and Food Safety Research Institute (INSA-UB), Santa Coloma de Gramenet, Spain.
- Carpio-Arias, T. Department of Nutrition, Food Science and Gastronomy, University of Barcelona, Bellaterra, Spain.
- Castell. M. Section of Physiology, Department of Biochemistry and Physiology, Faculty of Pharmacy and Food Sciences, University of Barcelona, Barcelona, Spain; Nutrition and Food Safety Research Institute (INSA-UB), Santa Coloma de Gramenet, Spain.
- Castellote, A.I. Department of Nutrition, Food Science and Gastronomy, Faculty of Pharmacy and Food Science, University of Barcelona, Barcelona, Spain; Institute for Research on Nutrition and Food Safety (INSA-UB), Barcelona, Spain; Institute of Health Carlos III, CIBER Physiopathology of Obesity and Nutrition (CIBEROBN), Madrid, Spain.
- Cherubini, A. Geriatrics and Geriatric Emergency Care, Italian National Research Centre on Aging (IRCCS-INRCA), Ancona, Italy.
- Chiva-Blanch, G. Cardiovascular Research Center (CSIC-ICCC), Barcelona, Spain; Department of Internal Medicine, Hospital Clinic, Institute of Biomedical Investigation August Pi i Sunyer (IDIBAPS), University of Barcelona, Barcelona, Spain.
- Chisaguano AM. Faculty of Health Sciences, Nutrition, University of San Francisco de Quito, Quito, Ecuador.
- Coll, X. Chocolates Amatller, Simon Coll, Barcelona, Spain.
- Colmán-Martínez, M. Department of Nutrition, Food Science and Gastronomy, XaRTA, INSA, School of Pharmacy and Food Science, University of Barcelona, Barcelona, Spain.
- Comas-Basté, O. Department of Nutrition, Food Science and Gastronomy, XaRTA, INSA, Faculty of Pharmacy and Food Science, University of Barcelona, Santa Coloma de Gramenet (Barcelona), Spain.
- Cordero-García, M. National Center of Food Science and Technology, Universidad de Costa Rica, Costa Rica.
- Corella, D. Department of Preventive Medicine and Public Health, University of Valencia, Spain; CIBER Physiopathology of obesity and nutrition (CIBEROBN), Institute of Health Carlos III, Madrid, Spain.
- Cornejo, T. Laboratory of Microbiology, Hospital Universitari Vall d’Hebron, Barcelona, Spain.
- Creus-Cuadros, A. Nutrition, Food Science and Gastronomy Department, Pharmacy and Food Science School, University of Barcelona, Av. Joan XXIII 27, Barcelona, Spain; CIBER Physiopathology of Obesity and Nutrition (CIBEROBN), Institute of Health Carlos III, Spain.
- Cuevas-Diarte, M.A. Section of Crystallography, Mineralogy and Mineral Deposits, Faculty of Earth Sciences, University of Barcelona, Barcelona, Spain.
- De Almeida, L. University of Barcelona, Faculty of Pharmacy and Food Science, Department of Nutrition, Food Science and Gastronomy, Barcelona, Spain; Institute for Research on Nutrition and Food Safety (INSA-UB), Barcelona, Spain.
- De Caria, S. Università degli Studi di Udine, Udine, Italy.
- De la Garza, A. Department of Nutrition, Food Science and Gastronomy, University of Barcelona- Faculty of Pharmacy and Food Science, Barcelona, Spain; Institute for Research on Nutrition and Food Safety (INSA-UB), Barcelona, Spain.
- De Simón, M. Laboratory of the Public Health Agency, Barcelona, Spain.
- Dinarès, M. Department of Nutrition, Food Science and Gastronomy-XARTA-INSA, School of Pharmacy and Food Science, University of Barcelona, Barcelona, Spain.
- Domínguez, A. CIBER Epidemiología y Salud Pública (CIBERESP), Instituto de Salud Carlos III, Madrid, Spain; Department of Public Health, University of Barcelona, Barcelona, Spain.
- Duelo, A. Department of Nutrition, Instituto Clínico del Déficit de DAO (ICDDAO), C/Pere i Pons 1, 08195 Sant Cugat del Vallès, Barcelona, Spain.
- Ekşi, H. Department of Biochemistry and Physiology, Faculty of Pharmacy and Food Sciences; Nutrition and Food Safety Research Institute-UB, University of Barcelona, Barcelona, Spain.
- Estruch, R. Department of Internal Medicine, Hospital Clinic, IDIBAPS, University of Barcelona, Bellaterra, Spain; CIBER Physiopathology of Obesity and Nutrition (CIBEROBN), Health Institute “Carlos III”, Madrid, Spain; Observatorio del Cacao.
- Farran-Codina, A. Department of Nutrition, Food Science and Gastronomy, XaRTA, INSA, Food and Nutrition Torribera Campus, Faculty of Pharmacy and Food Science, University of Barcelona, Santa Coloma de Gramenet, Spain.
- Ferrucci, L. Clinical Research Branch, National Institute on Aging, Baltimore, MD, USA.
- Fitó, M. Cardiovascular Risk and Nutrition Research Group (CARIN, Regicor Study Group), IMIM (Hospital del Mar Medical Research Institute), Barcelona, Spain; CIBER Fisiopatología de la Obesidad y Nutrición (CIBEROBN), Instituto de Salud Carlos III, Government of Spain.
- Franch, À. Section of Physiology, Department of Biochemistry and Physiology, Faculty of Pharmacy and Food Science, University of Barcelona, Barcelona, Spain; Nutrition and Food Safety Research Institute (INSA-UB), Santa Coloma de Gramenet, Spain.
- Garcia-Aloy, M. Biomarkers and Nutrimetabolomic Lab, Department of Nutrition, Food Sciences and Gastronomy, XaRTA, INSA, Faculty of Pharmacy and Food Science, Food and Nutrition Torribera Campus, University of Barcelona, Santa Coloma de Gramenet, Spain; CIBER Frailty and healthy aging (CIBERFES), Institute of Health Carlos III, Madrid, Spain.
- García-Guasch, M.T. Department of Nutrition, Food Science and Gastronomy, Faculty of Pharmacy and Food Science, Food and Nutrition Torribera Campus, University of Barcelona, Santa Coloma de Gramenet, Spain.
- Garrido, P. Department of Nutrition, Food Science and Gastronomy, Food and Nutrition Torribera Campus, Faculty of Pharmacy and Food Science, University of Barcelona, Santa Coloma de Gramenet, Spain.
- Gil, F. Museu de la Xocolata, Barcelona, Spain.
- Gómez-Catalán, J. Toxicology Unit, Department of Pharmacology, Toxicology and Medicinal Chemistry, Faculty of Pharmacy and Food Science, University of Barcelona, Barcelona, Spain.
- Gómez-Contreras, A. Department of Biochemistry and Physiology, Faculty of Pharmacy and Food Science; Nutrition and Food Safety Research Institute-UB, University of Barcelona, Barcelona, Spain.
- Grases-Pintó, B. Section of Physiology, Department of Biochemistry and Physiology, Faculty of Pharmacy and Food Science, University of Barcelona, Barcelona, Spain; Nutrition and Food Safety Research Institute (INSA-UB), Santa Coloma de Gramenet, Spain.
- Guerendiain, M.E. National University of Chimborazo, School of Medicine, Riobamba, Ecuador.
- Guo, X. Department of Nutrition, Food Science and Gastronomy, XaRTA, INSA-UB, School of Pharmacy and Food Science, University of Barcelona, Barcelona, Spain.
- Guix, S. Enteric Virus Group, Department of Microbiology, University of Barcelona, Barcelona, Spain; Nutrition and Food Safety Research Institute (INSA-UB), University of Barcelona, Santa Coloma de Gramanet, Spain.
- Haro, D. Department of Nutrition, Food Science and Gastronomy, Faculty of Pharmacy and Food Science, Food and Nutrition Torribera Campus, University of Barcelona, Santa Coloma de Gramenet, Spain.
- Hrvolová, B. Faculty of Science, University of Ostrava, Ostrava, Czech Republic.
- Hurtado-Barroso, S. Department of Nutrition, Food Science, and Gastronomy, School of Pharmacy and Food Science, INSA-University of Barcelona, Barcelona, Spain; CIBER Physiopathology of obesity and nutrition (CIBEROBN), Institute of Health Carlos III, Madrid, Spain.
- Illan, M. Nutrition, Food Science and Gastronomy Department, XaRTA, INSA, Pharmacy and Food Science School, University of Barcelona, Barcelona, Spain.
- Izquierdo-Casas, J. Department of Neurology, Hospital General de Catalunya, Sant Cugat del Vallès, Barcelona; Department of Basic Sciences, Universitat Internacional de Catalunya, Sant Cugat del Vallès, Barcelona, Spain.
- Izquierdo-Pulido, M. Department of Nutrition, Food Sciences, and Gastronomy, School of Pharmacy and Food Science, INSA-University of Barcelona, Barcelona, Spain; CIBER Physiopathology of obesity and nutrition (CIBEROBN), Institute of Health Carlos III, Madrid, Spain.
- Jauregui, O. Scientific and Technological Centers of the University of Barcelona (CCIT-UB), Barcelona, Spain.
- Juan, M.E. Department of Biochemistry and Physiology, Faculty of Pharmacy and Food Science; Nutrition and Food Safety Research Institute-UB, University of Barcelona, Barcelona, Spain.
- Kalina, J.; Faculty of Science, University of Ostrava, Ostrava, Czech Republic.
- Kundisova, I. Department of Biochemistry and Physiology, Faculty of Pharmacy and Food Science; Nutrition and Food Safety Research Institute-UB, University of Barcelona, Barcelona, Spain.
- Lamuela-Raventós, R.M. Department of Nutrition, Food Sciences, and Gastronomy, School of Pharmacy and Food Science, INSA-University of Barcelona, Barcelona, Spain; CIBER Physiopathology of obesity and nutrition (CIBEROBN), Institute of Health Carlos III, Madrid, Spain.
- Lara, E. CICITEX Junta de Extremadura, Badajoz, Spain.
- Latorre-Moratalla, M.L. Department of Nutrition, Food Sciences and Gastronomy, XaRTA, INSA, Faculty of Pharmacy and Food Science, University of Barcelona, Santa Coloma de Gramenet (Barcelona), Spain.
- Llobet, J.M. Toxicology Unit, Department of Pharmacology, Toxicology and Medicinal Chemistry, Faculty of Pharmacy and Food Sciences, University of Barcelona, Barcelona, Spain.
- Llorach, R. Biomarkers and Nutrimetabolomic Lab, Department of Nutrition, Food Sciences and Gastronomy, XaRTA, INSA, Food and Nutrition Torribera Campus, Faculty of Pharmacy and Food Science, University of Barcelona, Santa Coloma de Gramenet, Spain; CIBER Frailty and healthy aging (CIBERFES), Institute of Health Carlos III, Madrid, Spain.
- López-Sabater, M.C. Department of Nutrition, Food Science and Gastronomy, Faculty of Pharmacy and Food Science, University of Barcelona, Barcelona, Spain; Institute for Research on Nutrition and Food Safety (INSA-UB), Barcelona, Spain; Institute of Health Carlos III, CIBER Physiopathology of Obesity and Nutrition (CIBEROBN), Madrid, Spain.
- López-Tamames, E. Department of Nutrition, Food Sciences and Gastronomy, XaRTA, INSA, Food and Nutrition Torribera Campus, Faculty of Pharmacy and Food Science, University of Barcelona, Santa Coloma de Gramenet, Spain.
- Lorente-Gascón, M. Department of Basic Sciences, Universitat Internacional de Catalunya, Sant Cugat del Vallès, Barcelona, Spain.
- Luna, O. Department of Nutrition, Food Sciences and Gastronomy, Food and Nutrition Torribera Campus, Faculty of Pharmacy and Food Science, University of Barcelona, Santa Coloma de Gramenet, Spain.
- Martínez-González, M.A. Department of Preventive Medicine and Public Health, School of Medicine, University of Navarra, Pamplona, Spain; IdiSNA, Navarra Institute for Health Research, Pamplona, Spain; CIBER Fisiopatología de la Obesidad y Nutrición (CIBEROBN), Instituto de Salud Carlos III, Government of Spain.
- Medina-Remón, A. Department of Internal Medicine, Hospital Clínic, IDIBAPS, University of Barcelona, Barcelona, Spain; CIBER Fisiopatología de la Obesidad y Nutrición (CIBEROBN), Instituto de Salud Carlos III, Government of Spain.
- Marín-Morote, L. Section of Physiology, Department of Biochemistry and Physiology, Faculty of Pharmacy and Food Sciences, University of Barcelona, Barcelona, Spain.
- Marrero, P.F. Department of Nutrition, Food Sciences and Gastronomy, Faculty of Pharmacy and Food Science, Food and Nutrition Torribera Campus, University of Barcelona, Santa Coloma de Gramenet, Spain.
- Martinez, A. Department of Health, Government of Catalonia, Barcelona, Spain.
- Martinez, C. Department of Nutrition, Food Sciences and Gastronomy, Food and Nutrition Torribera Campus, Faculty of Pharmacy and Food Science, University of Barcelona, Santa Coloma de Gramenet, Spain.
- Martínez-Huélamo, M. Department of Nutrition, Food Science and Gastronomy, XaRTA, INSA, School of Pharmacy and Food Science, University of Barcelona, Barcelona, Spain; CIBER CB06/03 Physiopathology of Obesity and Nutrition (CIBEROBN), Madrid, Spain.
- Massot-Cladera, M. Section of Physiology, Department of Biochemistry and Physiology, Faculty of Pharmacy and Food Sciences, University of Barcelona, Barcelona, Spain; Nutrition and Food Safety Research Institute (INSA-UB), Santa Coloma de Gramenet, Spain.
- Mercader-Martí, M. Miguel Torres, Vilafranca del Penedés, Barcelona, Spain.
- Miranda, I. Department of Nutrition, Food Science and Gastronomy, Catalonian Reference Network on Food Technology (XaRTA), Nutrition and Food Safety Research Institute (INSA-UB), University of Barcelona, Food and Nutrition Torribera Campus, Santa Coloma de Gramenet, Spain.
- Miró, L. Section of Physiology, Department of Biochemistry and Physiology, Faculty of Pharmacy and Food Sciences; Nutrition and Food Safety Research Institute, University of Barcelona (UB), Barcelona, Spain.
- Montes, R. University of Santiago de Compostela, Nutritional Research and Analysis Institute, Santiago de Compostela, Spain; Institute of Health Carlos III, CIBER Physiopathology of Obesity and Nutrition (CIBEROBN), Madrid, Spain.
- Moreno-González, R. Department of Biochemistry and Physiology, Faculty of Pharmacy and Food Sciences; Nutrition and Food Safety Research Institute-UB, University of Barcelona, Barcelona, Spain.
- Moretó, M. Section of Physiology, Department of Biochemistry and Physiology, Faculty of Pharmacy and Food Sciences; Nutrition and Food Safety Research Institute, University of Barcelona (UB), Barcelona, Spain.
- Pereira, P. Instituto Superior de Ciências da Saúde Egas Moniz, Egas Moniz Cooperativa de Ensino Superior, Monte da Caparica, Portugal.
- Pérez-Bosque, A. Section of Physiology, Department of Biochemistry and Physiology, Faculty of Pharmacy and Food Sciences; Nutrition and Food Safety Research Institute, University of Barcelona (UB), Barcelona, Spain.
- Pérez-Cano, F.J. Section of Physiology, Department of Biochemistry and Physiology, Faculty of Pharmacy and Food Sciences, University of Barcelona, Barcelona, Spain; Nutrition and Food Safety Research Institute (INSA-UB), Santa Coloma de Gramenet, Spain.
- Pérez-Rodríguez, J.M. CICITEX Junta de Extremadura, Badajoz, Spain.
- Pintó, R.M. Enteric Virus Group, Department of Microbiology, University of Barcelona, Barcelona, Spain; Nutrition and Food Safety Research Institute (INSA-UB), University of Barcelona, Santa Coloma de Gramanet, Spain.
- Planas, J.M. Department of Biochemistry and Physiology, Faculty of Pharmacy and Food Sciences; Nutrition and Food Safety Research Institute-UB, University of Barcelona, Barcelona, Spain.
- Polo, J. APC Europe S.A., Granollers, Spain.
- Prieto, M.H. CICITEX Junta de Extremadura, Badajoz, Spain.
- Quifer-Rada, P. Department of Nutrition, Food Science and Gastronomy, XaRTA, INSA, School of Pharmacy and Food Science, Food and Nutrition Torribera Campus, University of Barcelona, Barcelona, Spain; CIBER Physiopathology of obesity and nutrition (CIBEROBN). Institute of Health Carlos III, Spain.
- Quintanilla-Casas, B. University of Barcelona, Barcelona, Spain.
- Rabanal, M. Section of Physiology, Department of Biochemistry and Physiology, Faculty of Pharmacy and Food Sciences, University of Barcelona, Barcelona, Spain; Nutrition and Food Safety Research Institute (INSA-UB), Santa Coloma de Gramenet, Spain.
- Rabassa, M. Biomarkers and Nutrimetabolomic Lab, Department of Nutrition, Food Sciences and Gastronomy, XaRTA, INSA, Food and Nutrition Torribera Campus, Faculty of Pharmacy and Food Science, University of Barcelona, Santa Coloma de Gramenet, Spain; CIBER Frailty and healthy aging (CIBERFES), Institute of Health Carlos III, Madrid, Spain.
- Relat, J. Department of Nutrition, Food Sciences and Gastronomy, Faculty of Pharmacy and Food Science, Food and Nutrition Torribera Campus, University of Barcelona, Santa Coloma de Gramenet, Spain.
- Rigo-Adrover, M.M. Section of Physiology, Department of Biochemistry and Physiology, Faculty of Pharmacy and Food Sciences, University of Barcelona, Barcelona, Spain Nutrition and Food Safety Research Institute (INSA-UB), Santa Coloma de Gramenet, Spain.
- Ríos-Hernández, A. Department of Nutrition, Food Science and Gastronomy, University of Barcelona, Barcelona, Spain.
- Riu-Aumatell, M. Department of Nutrition, Food Sciences and Gastronomy, XaRTA, INSA, Food and Nutrition Torribera Campus, Faculty of Pharmacy and Food Science, University of Barcelona, Santa Coloma de Gramenet, Spain.
- Rodriguez, R. Department of Basic Sciences, Faculty of Medicine and Health Sciences, International University of Catalonia, Sant Cugat del Vallès, Barcelona, Spain.
- Rossell, C. Department of Nutrition, Food Sciences and Gastronomy, Faculty of Pharmacy and Food Science, Food and Nutrition Torribera Campus, University of Barcelona, Santa Coloma de Gramenet, Spain.
- Rodríguez-Lagunas, M.J. Section of Physiology, Department of Biochemistry and Physiology, Faculty of Pharmacy and Food Sciences, University of Barcelona, Barcelona, Spain; Nutrition and Food Safety Research Institute (INSA-UB), Santa Coloma de Gramenet, Spain.
- Sabrià A. Enteric Virus Group, Department of Microbiology, University of Barcelona, Barcelona, Spain; Nutrition and Food Safety Research Institute (INSA-UB), University of Barcelona, Santa Coloma de Gramanet, Spain.
- Salas, I. University of Barcelona, Faculty of Pharmacy and Food Science, Department of Nutrition, Food Science and Gastronomy, Barcelona, Spain.
- Salas-Salvadó, J. Human Nutrition Unit, University Hospital of Sant Joan de Reus, Department of Biochemistry and Biotechnology, Faculty of Medicine and Health Sciences, IISPV, Rovira i Virgili University, Reus, Spain; CIBER Fisiopatología de la Obesidad y Nutrición (CIBEROBN), Instituto de Salud Carlos III, Government of Spain.
- Saldaña-Ruíz, S. Section of Physiology, Department of Biochemistry and Physiology, Faculty of Pharmacy and Food Sciences, University of Barcelona, Barcelona, Spain; Nutrition and Food Safety Research Institute (INSA-UB), Santa Coloma de Gramenet, Spain.
- Sánchez-Pérez, S. Department of Nutrition, Food Sciences and Gastronomy, XaRTA, INSA, Faculty of Pharmacy and Food Science, University of Barcelona, Santa Coloma de Gramenet (Barcelona), Spain.
- Sandoval, V. Department of Nutrition, Food Sciences and Gastronomy, Faculty of Pharmacy and Food Science, Food and Nutrition Torribera Campus, University of Barcelona, Santa Coloma de Gramenet, Spain.
- Sasot, G. Nutrition, Food Science and Gastronomy Department, Pharmacy and Food Science School, University of Barcelona, Av. Joan XXIII 27, Barcelona, Spain; CIBER Physiopathology of obesity and nutrition (CIBEROBN). Institute of Health Carlos III, Spain; INSA-UB, Nutrition and Food Safety Research Institute, University of Barcelona, Barcelona, Spain.
- Segura-Moreno, M. University of Granada, Centre of Excellence for Paediatric Research EURISTIKOS, Granada, Spain.
- Soler-Singla, L. Department of Neurology, Hospital General de Catalunya, Sant Cugat del Vallès, Barcelona; Department of Basic Sciences, Universitat Internacional de Catalunya, Sant Cugat del Vallès, Barcelona, Spain.
- Soriano, A. Department of Nutrition, Food Sciences and Gastronomy, Food and Nutrition Torribera Campus, Faculty of Pharmacy and Food Science, University of Barcelona, Santa Coloma de Gramenet, Spain.
- Termes-Escalé, M. Department of Nutrition, Food Sciences and Gastronomy, Food and Nutrition Torribera Campus, Faculty of Pharmacy and Food Science, University of Barcelona, Santa Coloma de Gramenet, Spain.
- Torner, N. Department of Health, Government of Catalonia, Barcelona, Spain; CIBER Epidemiología y Salud Pública (CIBERESP), Instituto de Salud Carlos III, Spain; Department of Public Health, University of Barcelona, Barcelona, Spain.
- Torres-Castro, P. Section of Physiology, Department of Biochemistry and Physiology, Faculty of Pharmacy and Food Sciences, University of Barcelona, Barcelona, Spain; Nutrition and Food Safety Research Institute (INSA-UB), Santa Coloma de Gramenet, Spain.
- Torres-Espínola, F.J. University of Granada, Centre of Excellence for Paediatric Research EURISTIKOS, Granada, Spain.
- Tran, C. Nutrition, Food Science and Gastronomy Department, XaRTA, INSA, Pharmacy and Food Science School, University of Barcelona, Barcelona, Spain.
- Tresserra-Rimbau, A. Department of Nutrition, Food Science and Gastronomy, XaRTA, INSA-UB, School of Pharmacy and Food Science, University of Barcelona, Barcelona, Spain; CIBER Physiopathology of obesity and nutrition (CIBEROBN), Institute of Health Carlos III, Madrid, Spain.
- Tresserras, J. Departament of History and Archaeology, Faculty of Geography and History, University of Barcelona, Barcelona, Spain.
- Urpi-Sarda, M. Biomarkers and Nutrimetabolomic Lab, Department of Nutrition, Food Sciences and Gastronomy, XaRTA, INSA, Food and Nutrition Torribera Campus, Faculty of Pharmacy and Food Science, University of Barcelona, Santa Coloma de Gramenet, Spain; CIBER Frailty and Healthy Aging (CIBERFES), Institute of Health Carlos III, Madrid, Spain.
- Valderas-Martínez, P. Department of Internal Medicine, Hospital Clinic, IDIBAPS, University of Barcelona; CIBER Physiopathology of Obesity and Nutrition (CIBEROBN),Health Institute “Carlos III”, Madrid, Spain.
- Vázquez-Fresno, R. Biomarkers and Nutrimetabolomic Lab, Department of Nutrition, Food Sciences and Gastronomy, XaRTA, INSA, Food and Nutrition Torribera Campus, Faculty of Pharmacy and Food Science, University of Barcelona, Santa Coloma de Gramenet, Spain.
- Veciana-Nogués, M.T. Department of Nutrition, Food Sciences and Gastronomy, XaRTA, INSA, Faculty of Pharmacy and Food Science, University of Barcelona, Santa Coloma de Gramenet (Barcelona), Spain.
- Vicente F. Instituto Superior de Ciências da Saúde Egas Moniz, Egas Moniz Cooperativa de Ensino Superior, Monte da Caparica, Portugal; Section of Physiology, Department of Biochemistry and Physiology, Faculty of Pharmacy and Food Sciences, University of Barcelona; Institute for Research on Nutrition and Food Safety (INSA-UB), Barcelona, Spain.
- Vichi, S. Department of Nutrition, Food Sciences and Gastronomy, University of Barcelona, Barcelona, Spain.
- Vidal-Carou, M.C. Department of Nutrition, Food Sciences and Gastronomy, XaRTA, INSA, Faculty of Pharmacy and Food Science, University of Barcelona, Santa Coloma de Gramenet (Barcelona), Spain.
- Zamora-Ros, R. Biomarkers group, Nutrition and Metabolism Section, International Agency for Research on Cancer, Lyon, France.
